# Gallium containing bioactive materials: A review of anticancer, antibacterial, and osteogenic properties

**DOI:** 10.1016/j.bioactmat.2021.12.034

**Published:** 2022-01-10

**Authors:** Fatih Kurtuldu, Nurshen Mutlu, Aldo R. Boccaccini, Dušan Galusek

**Affiliations:** a FunGlass, Alexander Dubček University of Trenčín, Študentská 2, 911 50, Trenčín, Slovakia; b Institute of Biomaterials, Department of Material Science and Engineering, University of Erlangen-Nuremberg, 91058, Erlangen, Germany; c Joint Glass Centre of the IIC SAS, TnUAD and FChFT STU, Študentská 2, 911 50, Trenčín, Slovakia

**Keywords:** Antibacterial, Bioactive materials, Cancer treatment, Hemostasis, Osteogenesis

## Abstract

The incorporation of gallium into bioactive materials has been reported to enhance osteogenesis, to influence blood clotting, and to induce anti-cancer and anti-bacterial activity. Gallium-doped biomaterials prepared by various techniques include melt-derived and sol-gel-derived bioactive glasses, calcium phosphate bioceramics, metals and coatings. In this review, we summarize the recently reported developments in antibacterial, anticancer, osteogenesis, and hemostasis properties of Ga-doped biomaterials and briefly outline the mechanisms leading to Ga biological effects. The key finding is that gallium addition to biomaterials has great potential for treating bone-related diseases since it can be efficiently transferred to the desired region at a controllable rate. Besides, it can be used as a potential substitute for antibiotics for the inhibition of infections during the initial and advanced phases of the wound healing process. Ga is also used as an anticancer agent due to the increased concentration of gallium around excessive cell proliferation (tumor) sites. Moreover, we highlight the possibility to design different therapeutic approaches aimed at increasing the efficiency of the use of gallium containing bioactive materials for multifunctional applications.

## Highlights

• Antibacterial, anticancer, osteogenesis, and hemostasis properties of Ga-containing biomaterials covered.
• The mechanisms of biological activity of Gallium are outlined.
• Applications in different fields of Ga-containing biomaterials prepared by various techniques are assessed.
• Recent trends in Ga containing bioactive glasses, calcium-phosphate bioceramics, and metallic alloys discussed.
• Future research directions of Ga-containing bioactive materials are discussed.

## Graphical abstract

**Figure:**
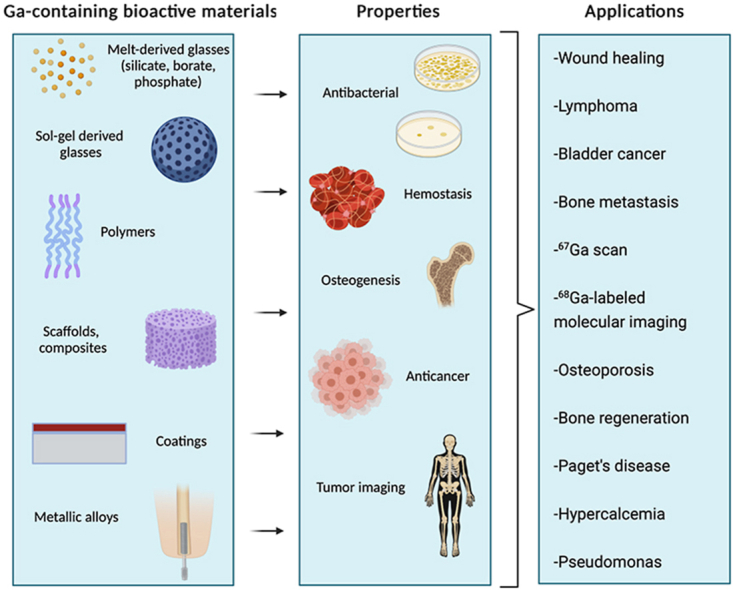

## Abbreviation list

**β-TCP**: β-tricalcium phosphate
**APTS**: 3-Triethoxysilylpropylamin
**ASCs**: Adipose-derived stem cells
**ASD**: Anodic spark plasma
**BAECs**: Bovine aortic endothelial cells
**BASMCs**: Bovine aortic smooth muscle cells
**BET**: Branauer-Emmett-Teller
**BGNs**: Bioactive glass nanoparticles
**CaP**: Calcium phosphate (Ca_3_(PO_4_)_2_)
**CDA**: Calcium-deficient apatite
**CMC**-Dex: Carboxymethyl cellulose and dextran
**CN**: Coordination number
**DMEM**: Dulbecco's modified eagle medium
**DSC**: Differential scanning calorimetry
**DTA-TG**: Differential thermal analysis-thermogravimetry
**EDX**: Energy dispersive x-ray
**EISA**: Evaporation induce self-assembly
**EPD**: Electrophoretic deposition
**EXAFS**: Extended x-ray absorption fine structure
**FDA**: U.S. food and drug administration
**F-FDG-PET**: Fluorodeoxyglucose - positron emission tomography
**FTIR**: Fourier-transform infrared spectroscopy
**GaP**: Gallium phosphate
**GPGs**: Gallium doped phosphate bioactive glasses
**HA**: Hydroxyapatite (Ca_10_(PO_4_)_6_(OH)_2_)
**HEXRD**: High energy X-Ray diffraction
**HRTEM**: High-resolution transmission electron microscope
**ICP-OES**: Inductively coupled plasma optical emission spectrometer
**LDHs**: Layered double hydroxides
**MBGs**: Mesoporous bioactive glass
**MBGNPs**: Mesoporous bioactive glass nanoparticles
**mBMSCs**: Mouse bone mesenchymal stem cells
**MMP-13**: Matrix metalloproteinase 13
**NBO**: Non-bridging oxygen
**NMR**: Nuclear magnetic resonance
**PBS**: Phosphate-buffered saline
**PCL**: Poly(ε-caprolactone)
**PDA**: Polydopamine
**PDLLA**: Poly-dl-lactic acid
**POC**: Poly(octanediol citrate)
**RANKL**: Receptor activator of nuclear factor- B ligand
**RF-MS**: Radio-frequency magnetron sputtering
**ROS**: Reactive oxygen species
**SBF**: Simulated body fluid
**SEM**: Scanning electron microscopy
**TEM**: Transmission electron microscopy
**TEOS**: Tetraethyl orthosilicate
**T**_**g**_: Glass transition temperature
**THB**: Todd Hewitt broth
**TRAP**: Tartrate-resistant acid phosphate
**VEGF**: Vascular endothelial growth factor
**XRD**: X-ray diffraction
**XPS**: X-ray photoelectron spectroscopy
**XRF**: X-ray fluorescence

## 1 Introduction

Metals have been used to fight a broad range of diseases from ancient civilizations to modern societies. Metallic ions addition to bioactive materials has been a subject of interest for the last few decades [[1], [2], [3], [4]]. The possibility of incorporating metallic ions dopants into bioactive materials has led to biomaterials with improved biological features to be tailored to specific clinical applications [1,[5], [6], [7], [8]]. For example, metallic ions like copper, strontium, zinc, silicon, boron, cerium, and gallium, usually incorporated in inorganic materials (e.g. in bioactive glasses and bioceramics), have emerged as potential therapeutic ions to enhance bone formation due to their ability to stimulate the expression of genes of osteoblast cells and to stimulate angiogenesis [1,2,9,10]. Furthermore, some therapeutic ions like silver, zinc, copper, cerium, and gallium have shown significant anti-bacterial and anti-inflammatory effects [[11], [12], [13], [14]]. This has also led to the development of antibiotic-free antibacterial agents exploiting antibacterial ion release [12].

**Fig. 1:**
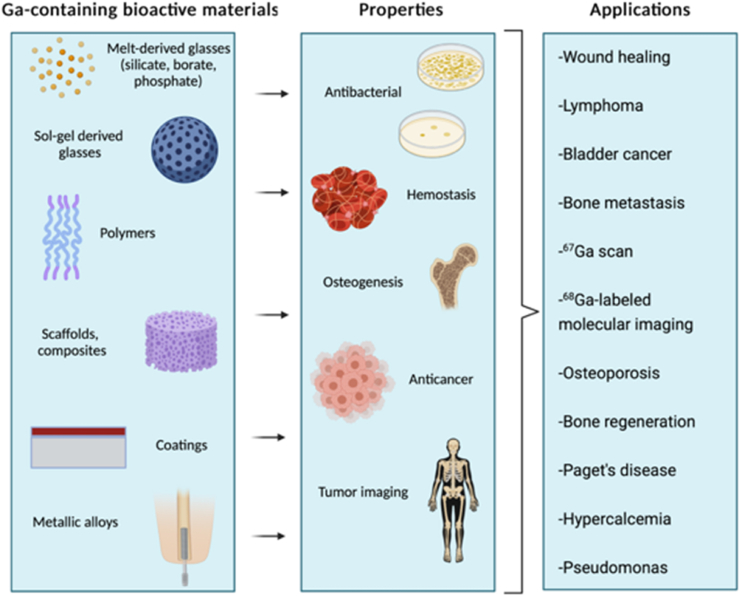
Biomedical application areas of gallium containing biomaterials (Created with BioRender.com).

Gallium is an important therapeutic ion for incorporation into bioactive materials. Gallium, a semi-metallic element in Group 13 of the periodic table, has shown a therapeutic effect for the treatment of numerous disorders. These could be categorized as follows; accelerated bone resorption, with or without elevated plasma calcium [15], autoimmune diseases and allograft rejection [16], hemostasis [17], and bacterial infections [[18], [19], [20]]. In addition to these therapeutic effects, gallium ions show antineoplastic activity against certain types of cancers [[21], [22], [23]]. These features set the gallium ions apart from other commonly used therapeutic ions. Fig. 1 summarizes the biomedical areas of application of gallium containing materials.

The use of gallium in the biomedical field was initiated by the 1950s' discovery that the radioactive isotope ^67^Ga, injected into rodents bearing implanted tumors, localized in high concentration within these tumors [24,25]. Due to this ability, the ^67^Ga isotope was used as a diagnostic tool for the detection of occult tumors or residual viable tumors following treatment in humans. Fluorodeoxyglucose - positron emission tomography (F-FDG- PET) scans have largely replaced the ^67^Ga scan in the last two decades [23], however, target-specific ^68^Ga labeled pharmaceuticals for molecular imaging are on clinical trials as advanced tools for PET studies [26,27]. ^68^Ga has a short half-life (t_1/2_ = 68 min) which enables rapid imaging [27]. Gallium was originally used only for imaging bone tumors, but in 1969, after the discovery of the ability of cumulation of ^67^Ga in soft tissue tumors, it became a useful tool for Hodgkin's disease treatment [28]. Initial studies suggested that all group 13 metals in the form of simple salts are capable of inhibiting tumor growth, but only gallium showed a therapeutic effect [29]. By the mid-1970s gallium nitrate had entered the clinical stages, and gallium became the second metal to show therapeutic activity in cancer patients after platinum [30]. The oral administration of gallium in the form of simple salts like gallium chloride allows continuous delivery of gallium ions. On the other hand, a combination of gallium with biomaterials improves the delivery of gallium directly to the affected area while minimizing the negative effect on healthy cells, as will be reviewed in this paper.

Gallium ions are included in a wide range of bioactive materials to induce multiple therapeutic effects over time. Gallium has a positive effect on bone cell growth [31]. Bone is a target organ for gallium; it accumulates within the bone and reduces calcium loss by inhibiting bone resorption without causing apparent damage to bone cells [32]. By observing the gallium distribution with synchrotron x-ray microscopy Bockman et al. [33] showed that gallium increased calcium and phosphorus content in bone, and also increased hydroxyapatite (HA) crystallites formation in maturing bone. Numerous studies report on the use of gallium salts for the treatment of bone loss such as osteoporosis, and bone metastases. However, when taken orally as a salt, the dose of Ga reaching the required bone site was low. An alternative way to administer gallium nitrate is a continuous intravenous infusion for 5–7 days. However, this method is inconvenient. As a more convenient alternative, gallium can be delivered to the required site in a controlled manner from melt-derived and sol-gel derived bioactive glasses and bio-ceramic-based scaffolds designed for bone tissue regeneration [34]. One of the most critical issues after surgery is bacterial infection associated to implanted biomaterials [35]. Gallium is also being tested in clinical trials to fight against infections [36,37]: gallium incorporation into coatings or scaffolds enhances the antibacterial properties of biomaterials [38]. Recently, gallium's hemostatic effects have been also examined [17,[39], [40], [41]]. The addition of gallium increased the capability of MBGs regarding platelet aggregation, thrombus formation, and blood coagulation activation [41]. Gallium addition to bioactive materials addresses some major issues related to the aging population, as different gallium-containing bioactive materials show reliable results for the treatment of numerous diseases, and current successful research results could progress to translation to the clinic.

This review is organized in the following way: section 2 summarizes the therapeutic and antibacterial activity mechanisms of gallium. Sections 3, 4, 5, 6 provide a comprehensive summary of various types of gallium containing bioactive materials in the following order: bioactive glasses, calcium phosphate bioceramics, coatings, and metallic alloys. Gallium's introduction to engineered biomaterials enables the development of a massive range of applications, from cancer treatment to wound healing. Finally, in section 7, the overall potential of gallium in biomaterials is discussed, and areas where future research is needed are identified.

## 2 Mechanisms of biological activity of gallium

Mechanisms of therapeutic activity and biochemistry of gallium have been studied and reviewed in several articles [15,22,23,[42], [43], [44]]. These subjects will be therefore reviewed here only briefly.

Gallium is a Group 13 metal element of the periodic table and only exists in the oxidation state +3. Ga^3+^ does not have any known essential role in the body, but it shares certain similarities with Fe^3+^. For example, the octahedral ionic radius is 0.62 Å in Ga^3+^, and 0.645 Å for high spin Fe^3+^. Also, the tetrahedral ionic radius is 0.47 Å in Ga^3+^, and 0.49 Å for high spin Fe^3+^ [45]. The ionization potential (4th ionization potential) values for Ga^3+^ and high spin Fe^3+^ are 64 eV and 54.8 eV, respectively. Electron affinity (3rd ionization potential) value for Ga^3+^ is 30.71 eV and 30.65 eV for high spin Fe^3+^ [15]. With these similarities, gallium can bond with iron-binding proteins. While the binding of iron to a protein promotes protein function, gallium, in contrast to iron, is not redox-active, so the substitution of gallium for iron in a protein usually disrupts its function and leads to negative downstream effects in cells [42,46,47].

**Fig. 2:**
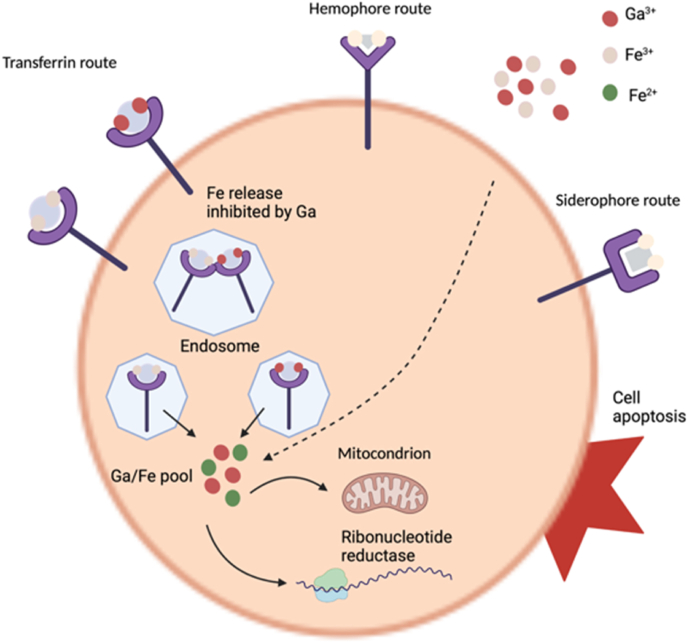
Schematic outline of anticancer activity of Ga. Fe and Ga are up-taken by the cells through transferrin and transferrin independent routes. Fe and Ga are unloaded in an acidic endosome and transfer to the pool, which can be inhibited by Ga. The Fe pool is used for ribonucleotide reductase and mitochondrial activity (Created with BioRender.com).

Besides platinum, gallium is a metal ion with anticancer properties. Despite the presence of contradictory studies, the therapeutic activity of gallium is, to a large extent, associated with the competition of Fe^3+^ and Ga^3+^ for cellular uptake [23]. The distribution of gallium is found to concentrate on proliferated tissues, including most tumors, due to a large amount of Fe^3+^ binding proteins [15]. The uptake system is thought to be associated with transferrin receptors which is illustrated in Fig. 2. In other words, highly proliferating tumor cells require more iron than normally dividing cells whereby, having a high concentration of receptors, they become an attractive target for gallium ions to bind to [15,48]. After gallium is taken into the cell, it binds to ribonucleotide reductase enzyme [49], which is responsible for DNA replication and repair, and prevents its activity, resulting in apoptosis through the mitochondrial pathway [50]. Since gallium is taken up by cancer cells in larger amounts than by normal cells, the normal cells are not negatively affected, but the viability of cancer cells decreases [51].

**Fig. 3:**
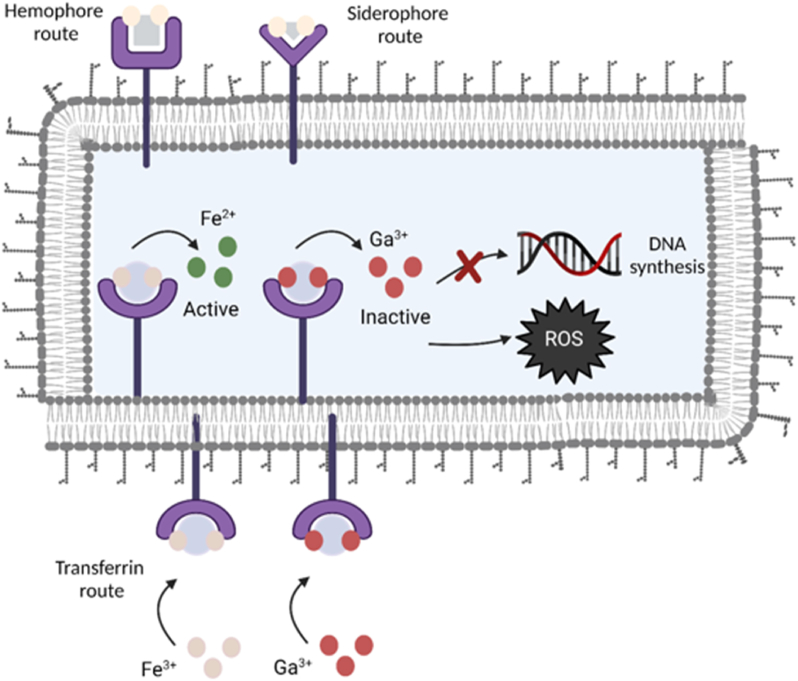
Schematic illustration of antibacterial activity of Ga. Ga crosses the cytoplasmic membrane of bacteria by using Fe-uptake routes using transferrin, homophora and siderophore. Ga cannot be reduced and critical functions such as DNA synthesis, respiration, and oxidative stress response are interrupted by Ga (Created with BioRender.com).

Iron is a key element in metabolic and signaling functions of bacteria due to its involvement in major biological processes, including cellular respiration, DNA synthesis, oxygen transport, and defense mechanism towards reactive oxygen species (ROS) [[52], [53], [54]]. During infection, bacteria are faced with a shortage of iron since the host reduces iron availability as a part of the immune system response to prevent the proliferation of bacteria [52]. Therefore, bacteria develop high-affinity ferric iron uptake mechanisms (illustrated in Fig. 3). One of them is the production of low molecular mass compounds called siderophores. A siderophore receptor is a small secreted iron-binding molecule that is part of the bacteria iron uptake system, along with a siderophore receptor protein, which actively transfers iron into the cell, allowing its solubilization and extraction [54]. Considering the chemical similarities between Fe^3+^ and Ga^3+^ [18], microorganisms cannot easily distinguish between these two ions and it has been hypothesized that bacteria sequester Ga through their iron uptake system since Ga has been shown to bind to iron siderophores [55]. Hereby, Ga^3+^ competes with Fe^3+^ for incorporation into essential proteins and enzymes. Unlike Fe^3+^, Ga^3+^ cannot be reduced under physiological conditions, resulting in inhibition of several iron-dependent redox pathways. However, some mutant strains of *P. aeruginosa* were reported to develop resistance against gallium administrated in the form of a simple salt, such as Ga(NO_3_)_3_ [[56], [57], [58], [59]]. The mechanism of such gallium resistance is not yet fully understood. The available literature suggests that the outflow mechanism of gallium from the bacteria may be responsible for the development of pathogen's resistance to gallium [57].

Besides its antibacterial and anticancer properties, many studies provide strong evidence of the osteogenic (anabolic) activity of gallium [15,60,61]. This activity is associated with a reduction in osteoclast activity and an increase in apoptosis-dependent cell death. Osteoclasts are multinucleated giant cells responsible for breaking down and resorbing bone tissue. They play an important role in liberating minerals and other molecules stored within the bone matrix. On the other hand, osteoblasts are responsible for building new bone tissue. The reduction of osteoclast activity is thought to be associated with an increase in the amount of calcium and phosphorus in bone tissue [15]. The postulated mechanism of Ga action is that it prevents the breakdown of the bone by blocking osteoclast activity, thus lowering the amount of free calcium in blood [62]. Gallium-treated bones showed an increased amount of calcium and phosphate content, which results in enhanced stability of bone associated with a larger size of HA crystals leading to higher resistance to bone resorption [63,64]. Bockman et al. proposed Ga uptake by HA, substituting it for calcium and altering the dissolution behavior of this phase [65].

Moreover, besides reducing the inflammation in the latter stage of wound healing, gallium also shows improvement during the very early stage of hemostasis - either coagulation, platelet activation, or clot formation [17,40,41,[66], [67], [68]]. This effect of gallium has been proven by comparing gallium containing composites with two different commercial products [69]. Although the exact mechanism is still unclear, studies indicate that gallium shows hemostasis capability with intrinsic coagulation pathway via activation of Factor XII in a similar manner to current commercial products [41,66,68,70].

## 3 Gallium in bioactive glasses

**Fig. 4:**
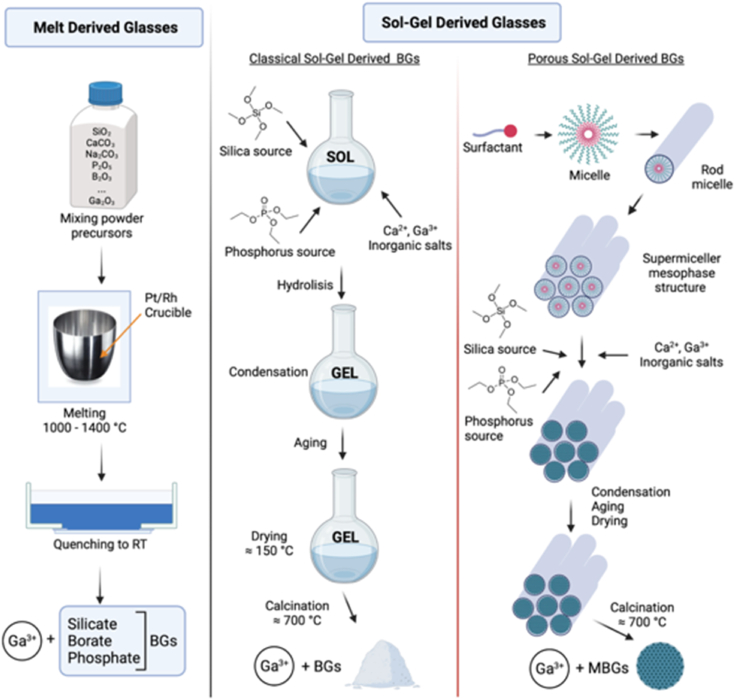
Illustration of the fabrication/synthesis of melt derived and sol-gel derived gallium containing bioactive glasses (Created with BioRender.com).

Bioactive and biodegradable glasses are a group of materials that can be used for hard as well as soft tissue engineering applications due to their wide range of compositions, morphologies and solubility, which can be tailored to the desired biological response [71,72]. The two main methods of synthesis of bioactive glasses are summarized in Fig. 4. This section describes the effect of gallium on the structure and properties of melt derived and sol-gel derived bioactive glasses. The glasses with incorporated gallium reported in the literature, their compositions, and investigated properties are briefly summarized in Table 1 and Table 2.

**Table 1:** Gallium containing melt derived bioactive glasses.

Composition (% mol)	Investigated Properties	Application Area	Features	Ref.
70SiO_2_-(30-x)CaO-xGa_2_O_3_ x = 2, 4%	Evaluation of gallium influence on glass structure by FTIR, NMR. Characterization of glass by XRF, XRD, DSC, SEM. In vitro ion release study in distilled water and SBF. In vitro biocompatibility and angiogenesis assay using MG-63. Evaluation of antibacterial activity against *E. coli* and *S. aureus*		Slightly increased cell viability. Improved VEGF secretion. Antibacterial activity.	[76]
42SiO_2_-(40-x)ZnO–10Na_2_O–8CaO-xGa_2_O_3_ x = 0, 8, 16%	Characterization of glass using XRD, particle size analysis, XPS, DTA. Structural evaluation by Raman spectroscopy and NMR. In vitro degradation study in ultra-pure water.		Predominantly network former, may exist in modifying role	[79,103]
46.2SiO_2_-24.3Na_2_O-26.9CaO-2.6P_2_O_5_-xGa_2_O_3_ x = 1.0, 1.6, 3.5	Glass characterization by XRD, ESEM, EDS. In vitro ion release and bioactivity study in SBF.		Bioactive response. Improved chemical durability.	[74]
45.7SiO_2_-24.1Na_2_O-26.6CaO-2.6P_2_O_5_-1.0Ga_2_O_3_	Functionalization with TEOS and APTS. In vitro degradation and bioactivity study in SBF. Characterization of functional groups with FTIR, ESEM-EDS.		Bioactive response	[81]
(46.1–3x)SiO_2_-26.9CaO-24.4Na_2_O-2.6P_2_O_5_-xGa_2_O_3_ x = 1, 2, 3%	Characterization of glass using SEM, XRD, FTIR, XRD. In vitro bioactivity evaluation in SBF. Dissolution study in ultra-pure water. Cell viability assay using human osteosarcoma (Saos-2) cells and primary normal human osteoblast	Bone cancer treatment	Decreased osteosarcoma viability. No effect on osteoblast viability	[86]
45P_2_O_5_–14CaO–35Na_2_O-3Ga_2_O_3_	Evaluation of antibacterial activity using *P. gingivalis*. In vitro MMP-13 activity assay. In vivo biocompatibility study using the Sprague Hypnorm rat model.	Periodontitis treatment	Lower MMP-13 activity	[101]
45P_2_O_5_-xCaO-(47-x)Na_2_O-3Ga_2_O_3_–5Ag_2_O x = 10, 11, 12	In vitro degradation test in deionized water. Evaluation of antibacterial activity and anti-biofilm properties using *P. aeruginosa*.	Combat infections	Antibacterial activity and anti-biofilm formation	[104]
P_2_O_5_–MgO–CaO–Na_2_O–Ga_2_O_3_–CeO_2_, up to 7% CeO_2_ or Ga_2_O_3_	Characterization of glass by Raman, XRD, FTIR, In vitro degradation study in deionized water. In vitro cell viability evaluation using bone marrow stromal cells (ST2). Evaluation of antibacterial activity against *E. coli* and *S. carnosus*.	Tissue engineering and wound healing	Less glass solubility. Decreased cell viability	[102]
45P_2_O_5_–16CaO-(39-x)Na_2_O-xGa_2_O_3_ x = 1, 3, 5	Degradation study in deionized water. In vitro antibacterial assay against *S. aureus, E. coli, P, aeruginosa*. Thermal and structural analysis via DSC, NMR, FTIR, Raman.	Bone tissue	Less glass solubility. Improved antibacterial activity	[97]
45P_2_O_5_-xCaO-(47-x)Na_2_O-3Ga_2_O_3_–5Ag_2_Ox = 10, 11, 12	Degradation study in deionized water. In vitro antibacterial assay against *P. gingivalis*, *S. gordonii* via oral biofilm model	Periodontal therapy	Improved antibacterial activity	[100]
(52-x)B_2_O_3_–16ZnO–14Na_2_O–12CaO–6P_2_O_5_-xGa_2_O_3_ x = 0, 2.5, 5, 10, 15 in wt. %	Characterization of glass by XRD, SEM, DSC. Structural influence evaluation by FTIR, Raman, NMR. In vitro ion release test in distilled water. Evaluation of antibacterial activity against *P. aeruginosa, S. epidermidis*. In vitro bioactivity and degradation study in SBF		Increase in ratio of BO_3_ to BO_4_, decreased glass solubility. Improved antibacterial activity	[89,92]
(52-x)B_2_O_3_–16ZnO–14Na_2_O–12CaO–6P_2_O_5_-xGa_2_O_3_ x = 0, 2.5, 5, 10, 15 in wt. %	In vitro degradation study in distilled water. Evaluation of cell viability using pre-osteoblast MC3T3-E1 and osteosarcoma SaOS-2 cells	Osteosarcoma related bone graft	Improved osteoblasts viability with lower Ga content. Decreased osteosarcomas viability using containing 5% wt. Ga_2_O_3_ glass extract for 7 days	[91]
B_2_O_3_–CaO–Na_2_O–K_2_O–MgO–P_2_O_5_–Ce_2_O_3_(1, 3, 5%)/Ga_2_O_3_(1, 5%)	Characterization of scaffold and powder by SEM-EDX, XRD, FTIR, DTA-TG. In vitro bioactivity and degradation studies in SBF. Determination of mechanical properties of sintered glass powder by Vickers microhardness test.	Bone tissue	Lower degradation rate. Bioactive response	[88]
B_2_O_3_–CaO–Na_2_O–K_2_O–MgO–P_2_O_5_–Ce_2_O_3_/Ga_2_O_3_/V_2_O_5_ up to 5 wt%	In vivo implantation into a connective tissue of subcutaneous area of rats. Evaluation of in vitro antibacterial activity against *S. aureus* and *E. coli*	Soft tissue	Lower angiogenesis potential. No antibacterial activity i*n vivo*.	[105]
48SiO_2_-(40-x)ZnO–12CaO-xGa_2_O_3_ x = 8, 16%	Characterization of glass by XRD, DTA, XPS. Evaluation of mechanical properties by compressive strength, biaxial flexural strength test.	Anti-cancerous bone cement	No significant change in T_g._ Lower compressive strength.	[82]
42SiO_2_–10Na_2_O–8CaO-(40-x)ZnO-xGa_2_O_3_ x = 0, 8, 16%	Characterization of glass using particle size and surface area analysis. Characterization of composite by SEM and EDAX. Determination of swelling characteristic of a hydrogel. In vitro ion release in PBS. In vitro cytotoxicity evaluation using L-929 mouse fibroblast and MC3T3-E1 human osteoblast	Bone void filling material	No significant change in cell viability up to 30 days incubation. Max. 4.7 mg/L Ga release within 30 days.	[106]
42SiO_2_–10Na_2_O–8CaO-(40-x)ZnO-xGa_2_O_3_ x = 0, 8, 16%	Characterization of composite via CP MAS-NMR, TEM, DSC. In vitro MG-63 osteosarcoma cell viability assay. In vitro ion release test in ultra-pure water.	Bone void filling material	Decreased osteosarcoma viability using obtained extracts from glasses and composites after 30 days	[51]
48SiO_2_–12CaO–32ZnO-8Ga_2_O_3_	Characterization of composite by SEM-EDX. In vitro ion release study in PBS and SBF. In vitro bioactivity evaluation in SBF. In vitro ion penetration test into bone tissue matrix. Antibacterial test against *E. coli* and *S. aureus* using liquid culture method.	Bone tissue	Delayed CaP precipitation. Improved antibacterial activity. Low concentration of Ga absorbed into bone.	[85]
42SiO_2_–10Na_2_O–8CaO-(40-x)ZnO-xGa_2_O_3_, x = 0, 8, 16%	Characterization of glass by particle size analysis, surface area analysis. In vitro degradation test in ultra-pure water and PBS. Antibacterial efficiency study against *E. coli, S. aureus, C. albicans*.	Bone void filling	Improved antifungal and antibacterial activity in viscous environment.	[38]
33SiO_2_-(18-x)-Al_2_O_3_–23CaO–11P_2_O_5_–15CaCl_2_ x = 12, 18 mol %	Characterization of alginate, glasses and composite by gel permeation chorography, NMR, FTIR, XRD, laser diffraction, DTA-TG, helium pycnometer, EXAFS, BET, XPS, FESEM, zeta potential. Evaluation of mechanical properties of composite by compression testing in cell medium. In vitro degradation study in DMEM. In vitro cytotoxicity evaluation using BASMCs and BAECs.	Cardiovascular tissue engineering	Matching stiffness with soft tissue. Slow and tunable gelation rate. No significant cell death. Increased wettability of glass. Prolonged working and hardening time. Increased strength after surface modification of glasses (acid washing).	[75,107]
B_2_O_3_–CaO–K_2_O–MgO–Na_2_O–P_2_O_5_1Ag_2_O/CeO_2_/CuO/Fe_2_O_3_/Ga_2_O_3_/SrO/Y_2_O_3_/ZnO in wt.%	In vitro degradation study in SBF. Evaluation of neuronal survival and neurite outgrowth in dorsal root ganglion from E11 chicks.	Peripheral nerve regeneration	Improved outgrowth of neurons and ratio of survival of neurons. Decreased survival of support cells	[94]
P_2_O_5_–CaO–MgO–Na_2_O–Ga_2_O_3_, up to 6 %Ga_2_O_3_	Evaluation of the structural influence of gallium addition by NMR, FTIR, micro-Raman. Mechanical properties by nano-indentation.	Orthopedic/dental implant	Improved mechanical properties.	[108]

**Table 2:** Gallium containing sol-gel derived bioactive glasses.

Composition (% mol)	Investigated Properties	Application Area	Features	Ref.
70SiO_2_-(30-x)CaO-xGa_2_O_3_ x = 2, 4%	Evaluation of gallium influence on glass structure by FTIR, NMR. Characterization of glass by XRF, XRD, DSC, SEM. In vitro ion release study in distilled water and SBF. In vitro biocompatibility and angiogenesis assay using MG-63. Evaluation of antibacterial activity against *E. coli* and *S. aureus*		Lower cell viability compared to melt derived counterpart. Improved VEGF secretion.	[76]
(80-x)SiO_2_–15CaO–5P_2_O_5_-x(Ce_2_O_3_/Ga_2_O_3_/ZnO) x = up to 7%	Characterization of glasses using NMR, XRD, FTIR, SEM, ICP. In vitro bioactivity in SBF.	Bone tissue	Reduction of glass network connectivity. Decreased bioactivity at high Ga content.	[77]
70SiO_2_–15CaO–10P_2_O_5_-5Ga_2_O_3_ 80SiO_2_–12CaO–3P_2_O_5_-5Ga_2_O_3_ 80SiO_2_–15CaO-5Ga_2_O_3_	Characterization of glass by XRD, EDX, BET, TEM, NMR. In vitro bioactivity test in SBF. In vitro degradation study in SBF, DMEM, and Todd Hewitt Broth culture medium.	Tissue engineering	Lower network connectivity and fast bioactive response with higher amount of modifier ions.	[31]
80SiO_2_–15CaO–5P_2_O_5_- 3.5Ga_2_O_3_/3.5CeO_2_/7ZnO	Characterization of glasses using XRD, TEM, BET, DTA-TG. In vitro bioactivity test in SBF.	Bone tissue	Decreased in mesopore order and textural properties. Bioactive response.	[114]
77.3SiO_2_-14.5CaO-4.8P_2_O_5_-3.4Ga_2_O_3_	Characterization of glasses via XRD, FTIR, ESEM, EDS, XPS, BET, CO adsorption, DMP Adsorption/Desorption. In vitro bioactivity and ion release test in SBF.	Bone tissue	Delayed bioactive response. Enhanced surface acidity. Slower glass dissolution	[115]
(80-x)SiO_2_–15CaO–5P_2_O_5_- (x = 0.2, 1.0)Ce_2_O_3_/Ga_2_O_3_/(x = 0.4, 2.0)ZnO	Characterization of scaffolds via XRD, BET, SEM, DTA-TG. In vitro bioactivity in SBF.	Bone tissue	Decreased surface area and pore volume. Bioactive response. Suitable pore structure	[124]
(80-x)SiO_2_–15CaO–5P_2_O_5_-xCe_2_O_3_/Ga_2_O_3_/ZnO x up to 4%	In vitro curcumin release test in SBF. Characterization of glasses by XRD, TEM, EDS, BET, DTA-TG. In vitro bioactivity in SBF.	Drug delivery	Exhibited suitable textural properties. Optimum drug loading and release at lowest Ga incorporation. Quick bioactive response.	[118]
(80-x)SiO_2_–15CaO–5P_2_O_5_-xGa_2_O_3_ x = 1, 2, 3 mol%	Characterization of glasses by BET, XRD, TEM, SEM-EDS, FTIR, zeta potential measurements. In vitro ion release and bioactivity test in Tris-HCl buffer solution. In vitro blood plasma coagulation assay. Absorption efficiency test in PBS. In vitro thrombus formation test. In vitro platelet adhesion test. Evaluation of antibacterial activity against *E. coli* and *S. aureus*. In vitro cytotoxicity assay using human dermal fibroblast.	Wound infection and hemostatic agent	Improved textural properties at lowest Ga content. Enhanced blood coagulation, thrombus generation, platelet adhesion and cell viability at lowest Ga content. Improved antibacterial activity.	[41]
58SiO_2_–31CaO–5P_2_O_5_-6Ga_2_O_3_ 85SiO_2_-8.4CaO–5P_2_O_5_-1.6Ga_2_O_3_	Characterization of glass by XRD, BET, XRF, NMR, TEM. In vitro bioactivity test in SBF. In vitro degradation study in MEM. In vitro cell viability, proliferation, early differentiation test using preosteoblast (MC3T3-E1) cells. In vitro osteoclast culture and viability test using the mouse monocyte cells (RAW 264.7)	Bone substitute in osteoporotic patients	Bioactive response. Decreased textural properties. Enhanced early differentiation of osteoblasts. Disturbed osteoclatogenesis.	[78]
(80-x)SiO_2_–15CaO–5P_2_O_5_-xGa_2_O_3_ x = 1%	Characterization of glass by FESEM, HRTEM, XRD, BET, Zeta sizer. Evaluation of water absorption capacity. In vitro degradation study in Tris-HCl. In vitro coagulation, thrombin generation, platelet adhesion, and thrombus formation assay. In vitro biocompatibility study using human dermal fibroblast cells. Hemostatic features compared with commercial products (Celox™ and QuicClot Advanced Clotting Sponge Plus™)	Hemostatic applications	Enhanced platelet adhesion. Improved contact activation (larger platelet aggregates, more extensive platelet pseudopodia). Accelerated clotting cascade. Increased cell viability.	[69]
77.3SiO_2_-14.5CaO-4.8P_2_O_5_-3.4Ga_2_O_3_	Characterization of glass by NMR, DTA-TG, XRD, HRTEM, BET, FTIR. Determination of drug release (curcumin) by UV–Vis. In vitro degradation test in SBF.	Drug delivery	Having textural properties to load large molecules (i.e., curcumin). Controlled drug release. Stabilization of Ga ions with curcumin. Local drug delivery.	[117]
80SiO_2_–15CaO–5P_2_O_5_ 3.5Ga_2_O_3_/3.5Ce_2_O_3_/7ZnO	Characterization of glass by XRD, BET, SEM, EDX. In vitro bioactivity test in SBF. Determination of the antibacterial activity of scaffold against *S. aureus*. In vitro biocompatibility using osteoblast-like cells	Bone tissue	Decreased textural properties and mesoporous order. Delayed bioactive response. No antibacterial activity.	[119]
70SiO_2_–15CaO–5P_2_O_5_-10Ga_2_O_3_	Glass characterization by XRD, EDX, FTIR, BET. In vitro degradation test in distilled water. Determination of mechanical properties by microhardness measurement. In vitro cell viability assay using human dental pulp stem cells	Orthodontic treatment	Decreased degree of enamel demineralization. Increased microhardness. No significant change of adhesive remnant index, cell viability and bacteria viability.	[121]
60SiO_2_-(40-x)CaO-xGa_2_O_3_ x = 1, 3 and 5 mol%	Glass characterization by SEM, BET, ICP-OES, XRD, FTIR. In vitro bioactivity test in SBF. In vitro degradation test in PBS. In vitro cell viability using MG-63. Antibacterial activity test against *S. aureus* and *E. coli*.	Drug delivery, bone tissue	Disorder mesoporous structure. Bioactive response. Slow release of Ga ions. Improved cell viability and antibacterial activity.	[123]

### 3.1 Gallium in melt-derived bioactive glasses

Melt derived bioactive glasses are produced by melting the precursors (oxides, carbonates etc.) in platinum crucibles at high temperature (between 1000 and 1400°), which depends on the composition of the bioactive glass [73]. Afterward, the glass melt is quenched in air or water to prevent crystallization, and the glass is produced (Fig. 4). Melt-derived glasses are classified based on their main network former oxide as silicate, phosphate, and borate bioactive glasses [72]. The effect of gallium on the structure and properties of silicate, phosphate, and borate bioactive glasses is comprehensively reviewed in this section.

#### 3.1.1 Silicate glasses

Silicate-based bioactive glasses are the most frequently studied and reported BGs in the literature. In the basic structural unit of the glass, silicon is surrounded by four oxygen atoms and forms SiO_4_ tetrahedra. The units can be bonded to a maximum of four other silica tetrahedra through their corner oxygen that establishes covalent bonds (Si–O–Si) between the tetrahedra. The silicate glasses contain network modifiers (Ca^2+^, Na^+^, K^+^) to break the continuity of the Si–O–Si bonds within the glass network, thereby forming non-bridging oxygens (Si-NBO). The presence of NBO leads to increasing ion exchange, and the rate of dissolution of the glasses is enhanced. Changing the chemical structure of the glass with additional elements is thus one of the important aspects to be considered in new glass composition designs as it will change the bioactivity and hydrolytic resistance of the glass.

**Fig. 5:**
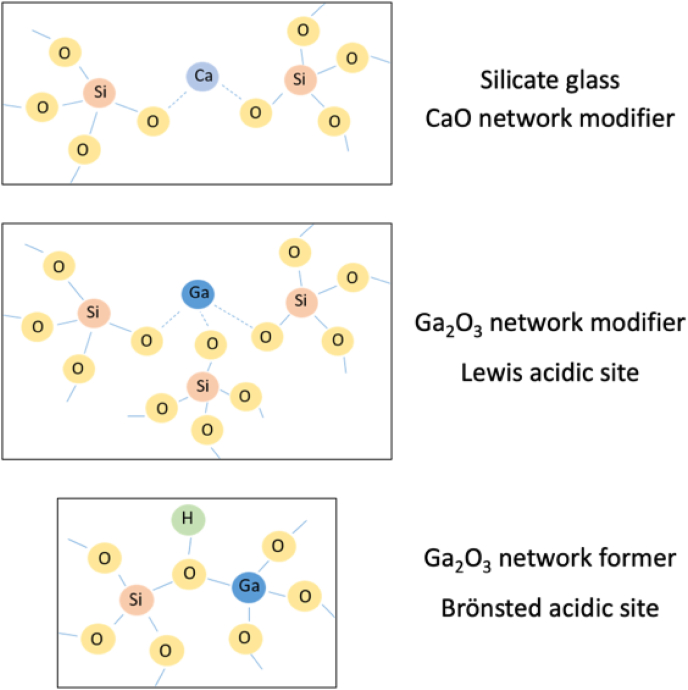
Effect of gallium in the silicate glass structure.

The addition of gallium has been shown to have a significant influence on the structural and thermal properties of bioactive glasses. When Ga is added into a silicate glass structure, it can act, similarly to Al^3+^, both as network former and network modifier [74,75] due to its ability to be incorporated in tetrahedral (coordination number CN = 4, GaO_4_) and octahedral (CN = 6, GaO_6_) structural units [[76], [77], [78]]. The studies show that Ga predominantly acts as a network former, however, a threshold level may exist where Ga starts to act as a network modifier [79]. With high amounts of alkali and alkali earth oxides in the glass composition, Ga^3+^ acts as a glass former, yet it could act as a glass modifier in high-silica glasses [31,80]. At low concentrations in the silicate system, Ga ions can act as network former, giving rise to Brönsted acidic sites with protonation of bridging oxygens of the Si–O-Ga groups (Fig. 5) [81]. When compared with other intermediate ions in the Si_2_O–CaO–P_2_O_5_ system such as Ce and Zn, Ga acts as an intermediate ion similar to Zn, while Ce mainly acts as network modifier [77].

The reduction of the number of Si–O covalent bonds increases the glass structure flexibility, which leads to a decrease of the glass transition temperature, T_g_, in the Ga-containing SiO_2_–CaO system [76]. The reduction of T_g_ is thus considered to be a consequence of a “weakened” glass network. However, it is suggested that the observed decrease of T_g_ in Ga-containing systems cannot be explained only by depolymerization of the glass network as it has been already proven that gallium creates Si–O-Ga bonds which have higher ionicity than Si–O–Si. The presence of these bonds leads to a reduction of directional covalent bonds and increases the flexibility of the glass structure that causes the reduction of T_g_ [76]. In contrast to this effect, theoretically calculated network connectivity increased from 1.83 to 2.5 and 2.7 with increasing Ga_2_O_3_ ratio in the SiO_2_–ZnO–CaO glass system from 0 to 8 and 16 mol % [82]. Wren et al. [79] also reported that in the SiO_2_–CaO–Na_2_O–ZnO-Ga_2_O_3_ melt-derived system, T_g_ shifted to higher temperatures (from 561 °C to 569 °C and 587 °C respectively) as the concentration of Ga in the glass melt increased from 0% to 8 mol % and 16 mol %. This shift is attributed to the formation of bridging oxygen groups (Si–O-Ga) which leads to an increase in glass stability [79].

The structural role of gallium as an intermediate element in the glass network is reflected in an increased chemical durability of Ga containing glass. This effect was observed when comparing the ability of 45S5 bioactive glass to form a HA layer during bioactivity tests [74]. Although 1 and 1.6 mol % gallium addition did not significantly affect this ability, incorporation of 3.5 mol % of gallium into the glass structure impaired the formation of HA. After incubation of 30 days in SBF, the surface of the glass was not covered homogenously with HA due to competition between Ca^2+^ and Ga^3+^ [74,83]. The ionic radius of Ga^3+^ (0.62 Å) is smaller than that of Ca^2+^ (0.99 Å) [60]. Therefore, Ga^3+^ can be incorporated into calcium phosphate (CaP) clusters more easily, interfering with their growth [60]. The highest concentration of Ga ions measured after 30 days exposition of 45S5 BGs in SBF was 6 ppm [74]. Higher gallium addition (8 and 16 mol %) caused a high Ga^3+^ release from the glass system (SiO_2_–CaO–Na_2_O–ZnO). The glass with 8 mol % of gallium showed the highest Ga release with 19.5, 26.8, and 37.4 ppm after 1, 7, and 14 days immersion in ultra-pure water, respectively [84].

Another point that should be considered is the biological response of gallium doped bioactive glasses towards different types of cells and bacteria, considering the application areas of these glasses. The antibacterial and antifungal efficiency of the Si_2_O–Na_2_O–CaO–ZnO glass system with the addition of up to 16% mol Ga_2_O_3_ and its carboxymethyl cellulose and dextran (CMC-Dex) hydrogel composites was examined against *S. aureus*, *E. coli,* and *C. albicans* [38]. Broth dilution and agar disc diffusion method were used for determining their antibacterial activity. In the broth dilution method, gallium doped glass and its composites showed an inhibitory effect towards *E. coli* and *C. albicans*. However, they did not show any inhibitory effect against *S. aureus.* Additionally, agar disc diffusion studies revealed higher antibacterial potential of gallium doped glass-hydrogel composites than suggested by the broth dilution tests: the results indicated that the antibacterial potential increased when the tested material was placed in fixed positions above and in direct contact with the material rather than in aqueous medium with bacteria seeded in the solution [38]. An agar disk diffusion test was performed also with a composite of the poly (octanediol citrate) (POC) matrix containing SiO_2_–CaO–ZnO glass with the addition of up to 3 wt % Ga_2_O_3_, examining its activity against *E. coli* and *S. aureus*. The antibacterial efficiency of the composite material increased with the concentration of the bioactive glass, and bacteria were inhibited significantly by the release of zinc and gallium ions [85].

Wajda et al. [76] performed an indirect in vitro biocompatibility and neovascularization study with SiO_2_–CaO glasses with the addition of 2 and 4 mol % gallium towards osteoblast-like cells. The number of attached cells and the mitochondrial activities of the cells showed an improvement compared to the control and the addition of gallium resulted in an increase in cell viability. Moreover, the gallium doped glasses showed an increase in vascular endothelial growth factor (VEGF) secretion and this effect was shown to be proportional to the Ga_2_O_3_ amount in the glass composition [76]. Clarkin et al. [75] produced injectable composite biomaterials with up to 18 mol % gallium oxide incorporated into a silicate glass (SiO_2_–CaO–P_2_O_5_–CaO–Al_2_O_3_) system as a substitution of alumina in an alginate hydrogel for cardiac tissue engineering applications. Gallium containing gels exhibited four times higher compressive strength than the minimum value required to withstand hypertensive blood pressure. Moreover, the addition of gallium prolonged working time which is needed for moving the material into suitable blood contact. Indirect test results indicated biocompatible properties of the prepared hydrogel towards Bovine aortic smooth muscle cells (BASMCs) and bovine aortic endothelial cells (BAECs) [75]. Another study conducted with CMC-Dex hydrogel containing SiO_2_–CaO–Na_2_O–ZnO-Ga_2_O_3_ glasses was aimed at the evaluation of the anti-cancerous property of gallium against MG-63 osteosarcoma cells. The extract of the glass containing 16 mol% gallium decreased the osteosarcoma cell viability to 79%, and the extract obtained from the composite with the highest amount of gallium decreased the viability to 69% after 30 days of incubation. Neither the bioactive glass itself nor the composite extracts diminished MC3T3-E1 osteoblast viability after the same time [51]. The difference is explained by the increase in iron metabolism. Cancer cells tend to divide rapidly and the uptake of gallium by cancer cells is proportional to their iron needs. This has a negative effect on the cancer cells viability in contrast to MC3T3-E1 osteoblast cells, whose iron (and gallium) intake is much lower [51]. A similar study was performed against osteosarcoma cells of a Saos-2 cell line [86]. 45S5 BG rods with the addition of up to 3 mol% Ga_2_O_3_ were used in the study. The cell viability decreased significantly with bioactive glass in a dose dependent manner. The Saos-2 cells’ viability was reduced to 50% after 72 h [86]. On the other hand, the glass positively affected normal human osteoblast cells, which showed good cell viability and proliferation in the same cell culture medium extract of gallium doped 45S5 bioactive glasses [76,86].

The discussed studies thus show that gallium containing silicate glasses possess suitable characteristics such as bioactivity (at a low amount of gallium addition) and antibacterial properties against Gram-positive and Gram-negative bacteria strains. In addition, they showed cytocompatibility along with improved vascularization potential as well as negative effects against cancer cells. Therefore, Ga containing silicate BGs are considered to have potential for a variety of biomedical applications.

#### 3.1.2 Borate glasses

The model for the structure of borate glasses differs significantly from that of silicate glasses. Boron occurs in both triangular and tetrahedral coordination. A small addition of alkaline oxide to the borate structure forces some of the boron to change from triangular to tetrahedral coordination, without formation of NBO. This change increases the connectivity of the network. After a critical concentration of tetrahedrally coordinated boron is achieved, more alkali oxide addition causes the formation of NBO which results in a reversal of the trend. This behavior is considered anomalous for glasses and is termed the boron anomaly. It also affects the glass transition, thermal expansion, and degradation behavior of the glasses [87].

Gallium addition could cause structural changes in borate bioactive glasses. For example, the incorporation of gallium to 1393-B3 BG composition resulted in the expansion of the sintering/processing window [88]. This happened through the replacement of the network modifiers with gallium, i.e. the replacement of Na^+^ and Ca^2+^ with Ga^3+^. The addition of network modifiers can widen the temperature interval in which the glass can be processed without crystallization, by increasing the activation energy of crystallization. The structural experiments were carried out in the system B_2_O_3_–Na_2_O–CaO–P_2_O_5_–ZnO. It is important to note that the compositions contained high amount of ZnO (16 wt%) and gallium was added as a substitution of boron. The results revealed that increasing gallium content in borate glass led to a decrease of the BO_4_/BO_3_ ratio which implies that the number of NBO is increasing [89]. However, contradictory results have been also reported in the literature. A study performed in the system B_2_O_3_–Rb_2_O_3_–Ga_2_O_3_, with Ga substituted for Rb, indicated that gallium preferentially bonded to B4 tetrahedra, and the relative representation of B3 species increased, lowering the percentage of B4's with increased gallium content [90]. According to Zachariasen's rules, oxygen can only be bonded to a maximum of two cations from the glass network, and the coordination number of that cation must be small (four or less). If gallium creates a tetrahedral unit in the glass network, the amount of oxygen in Ga_2_O_3_ is not sufficient to form GaO_4_ tetrahedra: it has to be, therefore, removed from the boron network. This separation may convert B4 to B3 with the increasing number of B3 species, lowering the content of B4 structural units [90]. This conversion may transform B4 into B3, meaning that gallium is acting more similarly to a network modifier. This also results in a change in the density of the glass. The density of borate glasses decreases with gallium addition, as the glass network makes space to accommodate gallium in the structure [90]. Another study performed in 1393-B3 composition demonstrated that the addition of gallium (1 and 5 wt%) showed higher Vickers' hardness compared to their gallium-free counterparts. This behavior is attributed to the formation of B4 groups since B4 groups are more strongly bonded than B3 groups, resulting in a more compact structure [88].

The structural role of Ga in borate glasses is not clear. The studies in the literature cannot often be compared due to large differences in studied compositions, as well as different approaches followed in gallium addition. To date, only a limited number of structural studies on the addition of gallium into borate glass has been identified, and thus the mechanisms that underpin the effect of gallium on the glass structure are not fully understood.

These structural changes are also reflected when considering differences in the rate of dissolution of borate bioactive glasses. If Ce_2_O_3_ and Ga_2_O_3_ were added into the 1393-B3 glass system simultaneously, the rate of dissolution would be reduced. Despite the slowdown in the release of ions, the bioactive responses of borate bioactive glasses were not affected when immersed in SBF [88]. The slower release of ions could be explained by the fact that gallium is part of the glass network alongside borates. Besides, gallium can be neutralized with (BO_3_)^3-^ species which makes the release of gallium more difficult, when compared with the other ions from the glass network [88].

A borate glass system (B_2_O_3_–Na_2_O–CaO–P_2_O_5_–ZnO-Ga_2_O_3_) incorporating up to 16 wt % of gallium was investigated by Yazdi et al. [91]. Degradation rate and weight loss of the glass in deionized water were reduced with gallium addition: it followed to changes in the glass network, where gallium substituted boron [92]. No crystalline phase including HA was detected after 28 incubation days in SBF, suggesting that zinc inhibited the precipitation of HA [92]. Ga was also found to have an inhibitory effect on HA formation and growth in a Ca-containing solution [93]. This phenomenon could be explained by two different mechanisms: substituting Ca^2+^ with Ga^3+^, which prevents the transformation to HA, and rejection by the HA layer with possible adsorption of Ga ions on the surface of HA crystals. In the latter case, gallium ions are not incorporated into the already formed HA layer. Instead, they might be loosely bound to the surface of the HA crystal and interfering with their further growth [93]. Therefore, it could be speculated that gallium, as well as zinc, could be the reason for preventing the formation of HA in such borate glass systems.

The biological response of gallium doped borate glasses has also been investigated [91,92]. Gallium addition in the B_2_O_3_–Na_2_O–CaO–P_2_O_5_–ZnO glass system was shown to improve the antibacterial properties of the glass significantly. A considerably higher inhibition against *P. aeruginosa* (gram-negative) was observed with the increase of gallium amount for up to 28 days incubation, while no visible inhibition was observed in the gallium free composition. On the contrary, the inhibitory zone diameter was diminished with a proportion of gallium amount in the glass against *S. epidermidis* (gram-positive). This effect could be explained by the cell wall differences between bacteria [92]. An increase of cell viability up to 10 wt % was observed in the same glass system using the dissolution products from the glasses. However, the extract of glass containing 15 wt % of Ga_2_O_3_ surpassed the toxic level for pre-osteoblasts after incubation of 7 and 28 days. The most effective zinc borate glass composition promoting the viability of osteoblasts and suppression of osteosarcoma cells was the system with 5 wt % Ga_2_O_3_ [91].

A comprehensive study was conducted with 1393-B3 glasses doped with various ions such as Ag, Zn, Ga and Ce shaped as rods with varying diameters, 50–200 μm, for nerve tissue regeneration. The gallium doped borate glass rods were embedded into poly(ε-caprolactone) (PCL) films. They significantly increased the survival of neurons, but compared to undoped glass the survival of support cells in response to gallium was significantly reduced after 10 days [94].

Deliormanlı et al. [95] studied photoluminescence and decay characteristics of gallium containing 1393-B3 glass powders for non-destructive in-vivo bioimaging applications. With Ga doped glass excited around 360 nm, optical band gap energies were found to be 3.44 eV, and the glass showed broadband green emission centered at 440 nm. Also, it exhibited large Stokes shifts and bi-exponential decays in nanosecond and microsecond time scales. Stokes shift is critical to the high sensitivity of fluorescence imaging and the existence of different fluorescence excited-state lifetimes can overcome the problem for the identification of different fractions. Therefore, gallium doped borate glasses could have an advantage for bioimaging applications [95].

Summarizing, results show that although the incorporation of gallium into borate glass systems leads to slower ion release, the addition of a low amount of gallium does not affect the bioactivity. Gallium doped borate glasses reported in the literature have shown biocompatible response, which is essential for biological applications, in a dose-dependent manner. Moreover, such glasses were shown to inhibit the growth of gram-negative bacteria. Some gallium-doped borate bioactive glasses possess photoluminescence properties that could be utilized for bioimaging applications.

#### 3.1.3 Phosphate glasses

The structure of phosphate glasses is also based on tetrahedral building blocks. In a pure phosphate system, the glass structure consists of a three-dimensional network with three bridging oxygens and one double-bonded oxygen. The model describing silicate glasses is applicable also for the incorporation of modifier ions in the phosphate glass system. Additional alkaline or alkaline earth oxides cause the breaking of (P–O–P) rings and the conversion of the network to a system of entangled linear chains of phosphorous-oxygen tetrahedra crosslinked by monovalent or divalent ions [87].

Valappil et al. [96] studied the addition of 1–3 mol% of Ga_2_O_3_ into CaO–Na_2_O–P_2_O_5_ glass. The addition of 3 mol% of Ga_2_O_3_ increased the glass transition temperature (T_g_) from 327 ± 1.2 °C to 343.3 ± 2.0 °C. This effect was confirmed in a similar glass system (P_2_O_5_–CaO–Na_2_O) with up to 5 mol% of Ga_2_O_3_ addition [97]. These changes are attributed to the formation of more ionic crosslinking within the phosphate glass network with the addition of Ga_2_O_3_. According to ^31^P MAS NMR results, the addition of Ga showed a slightly higher percentage of Q^2^ units than the glass without gallium and no Q^3^ or Q^0^ sites were observed. This suggests that with the presence of gallium, the network undergoes some slight rearrangements increasing the network connectivity of the glasses via creating relatively strong covalent Ga-O-P bonds [98]. This is supported by the fact that the ^71^Ga NMR peak is observed mostly in octahedral coordination [96]. High energy X-Ray diffraction (HEXRD) results have indicated that Ga^3+^ ions could be included in the phosphate glass network decreasing the chain length [99]. The addition of gallium into the phosphate glass system then leads to more chemically durable glasses [97].

Along with the structural effect, the incorporation of gallium into different phosphate glass compositions has been shown to influence the formation of a HA surface layer and the dissolution profile of the ions released from the glass. Valappil et al. [97] observed that the increasing addition of Ga_2_O_3_ reduced the dissolution rate of the phosphate glass system. Ga-free glass was found to dissolve completely after 72 h of exposure to deionized water. Both Na^+^ and Ca^2+^ ion release showed descending trend with increasing amount of Ga_2_O_3_. The highest level of Ga^3+^ ions was released from the glass with the lowest amount of Ga_2_O_3_ addition [97]. Moreover, the release of gallium ions from phosphate glasses can be controlled by changing the content of calcium: the solubility of gallium decreased with an increasing amount of CaO due to an increase in the ionic strength of the leaching solution [96].

In order to investigate the antibacterial properties of Ga-containing phosphate glasses, melt-derived silver and gallium doped phosphate glasses were tested in terms of the inhibition of biofilm formation against *Porphyromonas gingivalis*, periodontal pathogen, and *S. gordonii*, pioneer colonizer, for periodontal therapy [100]. According to the study, the simultaneous release of Ag and Ga considerably reduced biofilm formation of *P. gingivalis* after 7 days of exposure. The studied glass system offers an effective alternative to the use of antibiotics for infected sites in the oral cavity [100]. Other studies [97,101] confirmed an inhibitory effect of phosphate glasses containing 1 and 3 mol% of gallium against Gram-negative and Gram-positive bacteria.

An in vitro cytotoxicity study was performed with ST-2 cells using P_2_O_5_–MgO–CaO–Na_2_O glasses with added CeO_2_ and/or Ga_2_O_3_. Although the undoped glass showed cytocompatibility, up to 7 mol% gallium additions resulted in toxic behavior due to the high release of gallium. It has been pointed out that a glass co-doped with Ce and Ga could be a promising candidate for tissue engineering and wound healing applications since it showed strong antibacterial activity and improved biocompatibility compared to the high gallium containing glass compositions [102].

In vivo evaluation of the biocompatibility of the P_2_O_5_–Na_2_O–CaO system with gallium addition has been also performed in a rat model. The glass discs were implanted in the abdominal region and an initial response was observed. The results showed that gallium containing glasses were comparable with their gallium free counterpart. However, the in vivo response was more prominent for the gallium doped composition. It was assumed that higher calcium content and a corresponding decrease in degradation rate lead to the inflated immune response for gallium containing glass [101]. Moreover, enzyme assays demonstrated that the activity of matrix metalloproteinase 13 (MMP-13), which is capable of degrading a variety of extracellular matrix (ECM) components, was reduced when treated with gallium containing glass [101].

The discussed studies show thus a downregulated trend in ion release with the addition of gallium to phosphate glass systems. The systems with low amounts of incorporated Ga exhibit antibacterial activity, inhibiting biofilm formation and non-toxic response in vitro. Although in vivo studies showed inflammatory reactions against gallium containing glass, this response could be avoided by tuning the glass composition.

### 3.2 Gallium in sol-gel derived bioactive glasses

Li et al. first introduced bioactive glasses prepared by sol-gel process in 1991 [109]. Gel-derived glasses exhibit many advantages over melt-derived glasses, such as increased surface area, nano porosity, purity, and reduced processing temperature. Additionally, sol-gel derived glasses could be produced in many varieties, and bioactive glasses can be synthesized as solids, powders, or nanoparticles by changing the pH during synthesis [110]. Bioactive glass nanoparticles (BGNs) are promising materials to be used as a filler in composites, especially in polymer matrices [2,111]. Furthermore, with the addition of structural directive agents, bioactive glasses can be prepared with complex morphologies, including mesoporous structures [112]. BGs with mesoporous structure lead to the development of new classes of bioactive materials which can act as carriers for drugs and other biomolecules. Conventional melt-derived bioactive glasses show bioactivity in a narrow range of compositions. When a glass composition contains 60 mol% SiO_2_ or more, the glass does not show bioactivity, and bonding to soft and hard tissues is no longer observed. Sol-gel derived glasses show bioactivity with a broad range of compositions. Martinez et al. [113] studied sol-gel derived glasses in the CaO–SiO_2_ binary system with up to 90 mol% SiO_2_, which still showed bioactivity. These glasses could take phosphorus from a medium like SBF to form an HA-like phase on their surface. It is important to specify that the release of Ga^3+^ from bioactive glasses does not directly depend on the glass composition, but on the role of Ga in the glass network [114]. The incorporation of Ga into melt-derived silicate glass generally indicates Ca^2+^ versus Ga^3+^ ionic exchange. On the other hand, Gómez-Cerezo et al. [78] showed that incorporation of Ga into sol-gel derived mesoporous bioactive glasses (MBGs) is not only related to a Ga/Ca ion exchange process, but also to the formation of tetrahedral units as a network former, and octahedral units as a network modifier via Ga-O-Si covalent bond. Salinas et al. [114] reported that without Ga doping, MBGs exhibit ordered hexagonal meso-structure in SiO_2_–CaO–P_2_O_5_ glass. With the addition of up to 3.5 mol% Ga_2_O_3_ into the glass, the mesoporous order was decreased. A higher addition of Ga_2_O_3_ (from 3.5 to 5 mol%) did not cause a further change of textural properties [114].

Aina et al. [115] investigated the relationship between surface chemical properties and the bioactive response of Ga-modified (Si–Ca–P) sol-gel glasses. Even if the modified glass positively responded to the bioactivity test in SBF, a severe delay in apatite deposition/crystallization was observed, compared with the parent glass, due to the changes induced by the presence of Ga_2_O_3_ in the glass composition [115]. Similarly to melt-derived silicate glasses, Ga is acting as both network modifier and former, causing the accumulation of acidic sites at the surface of the glass [115]. The replacement of lower valence ions for silicon increases the surface acidity of the glass which inhibits HA crystallization. The negative charge enters the silicate glass system with Ga/Si substitution compensated by a proton and creates a Brönsted acid site (see Fig. 5) in the form of [Si(OH)^+^Ga^−^]. This protonic acidity increases the acidity of the surface of the glass [115]. The presence of both Brönsted and strong Lewis acid sites initially inhibits the deposition of Ca-phosphate [115]. It has been demonstrated that increasing surface acidity plays an important role in the bioactivity response [115]. Shruti et al. [77] also documented that the addition of up to 2 mol% Ga_2_O_3_ in silicate glass slightly delayed the formation of HA. Further increase of the Ga_2_O_3_ concentration (>2 mol %) in the glass caused a significant reduction in the bioactive response after up to 15 days of SBF immersion [77]. Surprisingly, the co-precipitation of Ga species (either Ga phosphate or Ga oxide), which according to the literature [116] delays/partially inhibits precipitation of CaP, was not confirmed. Moreover, modification of Si–Ca–P sol-gel glasses with Ga makes them suitable for interaction with biomolecules such as proteins or drugs due to the improvement of Lewis acidic strength [115]. Malavasi et al. [117] studied MBGs containing 3.4 mol% of gallium (77.3SiO_2_-14.5CaO-4.8P_2_O-3.4Ga_2_O_3_) prepared by the evaporation induced self-assembly (EISA) method. The presence of Ga^3+^ in the MBGs increased the number of uploaded drug molecules and also slowed down their release in the biological medium [118]. The presence of Ga_2_O_3_ in the Si–Ca–P glass composition also reduced its micro porosity. The reduced volume of micropores and surface area are believed to be responsible for delaying the growth of the apatite-like layer in the SBF medium [115].

Salinas et al. [114] doped 80SiO_2_–15CaO–5P_2_O_5_ MBGs with up to 3.5 mol % of gallium, replacing SiO_2_ in the glass composition with the aim of improving the mechanical properties of newly formed bone with gallium addition. The gallium doped MBGs maintained the mesoporous structure and textural properties characteristic of the undoped MBGs. In addition, the MBGs also showed in vitro forming ability of apatite-like phases. An initial formation of a calcium phosphate layer was observed after 6h in SBF: the morphology changed significantly after 1 day exposure. The entire glass surface was covered with an apatite-like layer [114].

**Fig. 6:**
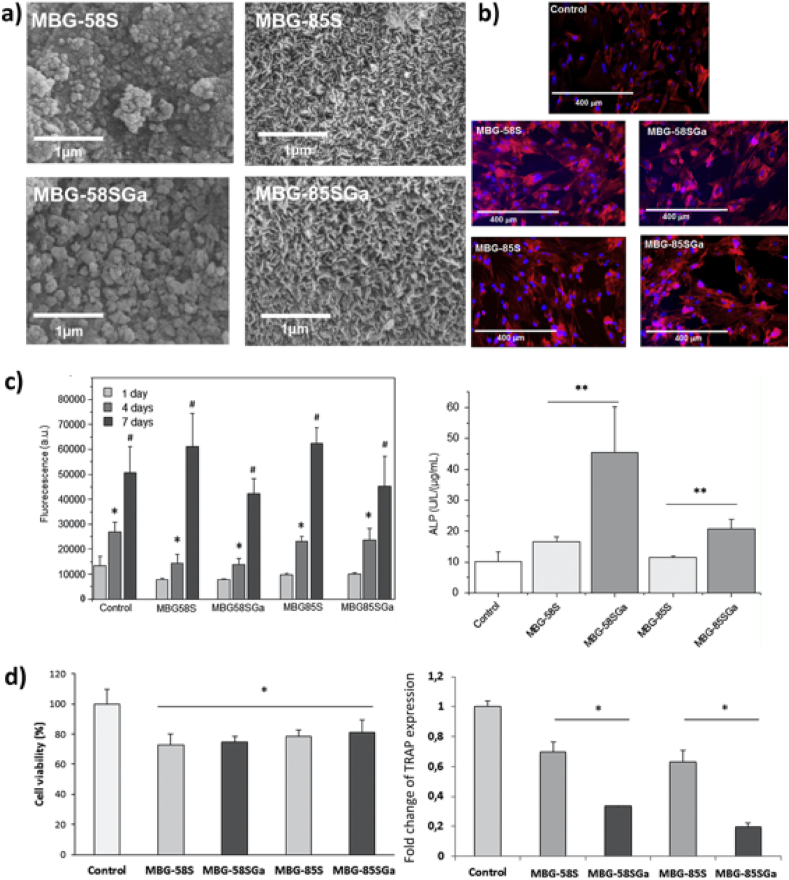
a) SEM images ofMBGs after immersion in SBF for 7 days, b) fluorescence microscopy images of MC3T3-E1 preosteoblast like cells after direct contact with MBGs for 4 days, c) proliferation of MC3T3-E1 cells in direct contact with MBGs after 1, 4 and 7 days incubation (*p < 0.05, #p < 0.01) and their ALP activity after 7 day incubation with MBGs (**p < 0.05), d) RAW 264.7 mouse monocytes viability after 4 days incubation with direct contact of 10 mg/mL *MBGs in the presence of* 20 nM RANKL and effect of MBGs on the TRAP expression of mature osteoclasts (normalized results corresponding to the control, *p < 0.05). Reproduced with permission from ref. [78]. *Copyright 2018 Elsevier.*

The antibacterial properties of Ga doped SiO_2_–CaO–P_2_O_5_ MBGs against *S. aureus* have been also investigated. The release of Ga^3+^ ions from the glass network was limited and no antibacterial effect of MBGs was confirmed [119]. Three gallium enriched MBGs with the composition xSiO_2_-yCaO-zP_2_O_5_-5Ga_2_O_3_ (mol %), where x = 70, y = 15, z = 10 for Ga_1; x = 80, y = 12, z = 3 for Ga_2; and x = 80, y = 15, z = 0 for Ga_3 were also studied [31]. The results of MAS NMR confirmed that Ga^3+^ ions acted mainly as network modifiers. Ga_1 showed the highest bioactivity due to the higher concentration of modifier ions and a more depolymerized network. After 3 days of exposure to SBF, the surface of Ga_2 was completely covered with HA. No HA was detected on the surface of Ga_3 even after 7 days due to the phosphorous-free composition. The release of Ca and Ga was also related to the P_2_O_5_ content: the glass with lower P_2_O_5_ content released Ca^2+^ ions faster [31]. Although the authors did not perform biological tests, Ga_1 was able to release Ga^3+^ ions to act effectively against *P. aeruginosa* and *S. aureus*. After 7 days of incubation, the amounts of Ga^3+^ ion released were 2.5 ppm and 9.8 ppm in DMEM and Todd Hewitt Broth (THB), respectively. The release amount in DMEM (2.5 ppm) was also below the toxicity limit in blood plasma, and the released amount in THB (9.8 ppm) was in the range of efficacy for antibacterial inhibition. Additionally, Cerezo et al. [78] studied the effect of Ga incorporation in SiO_2_–CaO–P_2_O_5_ MBGs with different amounts of SiO_2_ on pre-osteoblasts and osteoclasts. Whereas the Ga-containing MBGs showed a good proliferation behavior and significantly higher ALP activity, the TRAP expression from mature osteoclasts (RANKL induced RAW 264.7) decreased significantly with respect to Ga free MBGs (Fig. 6). These results suggest that gallium exhibits a selective behavior towards different cells: it enhances the early differentiation of osteoblast cells while disturbing osteoclast differentiation [78]. Ciraldo et al. [120] took advantage of these Ga doped MBGs properties and applied them to coat 45S5 BG scaffolds based on natural marine sponges. Ga doped MBGs added to the scaffolds led to good biocompatibility with MC3T3-E1 pre-osteoblastic cells and to additional antibacterial properties.

**Fig. 7:**
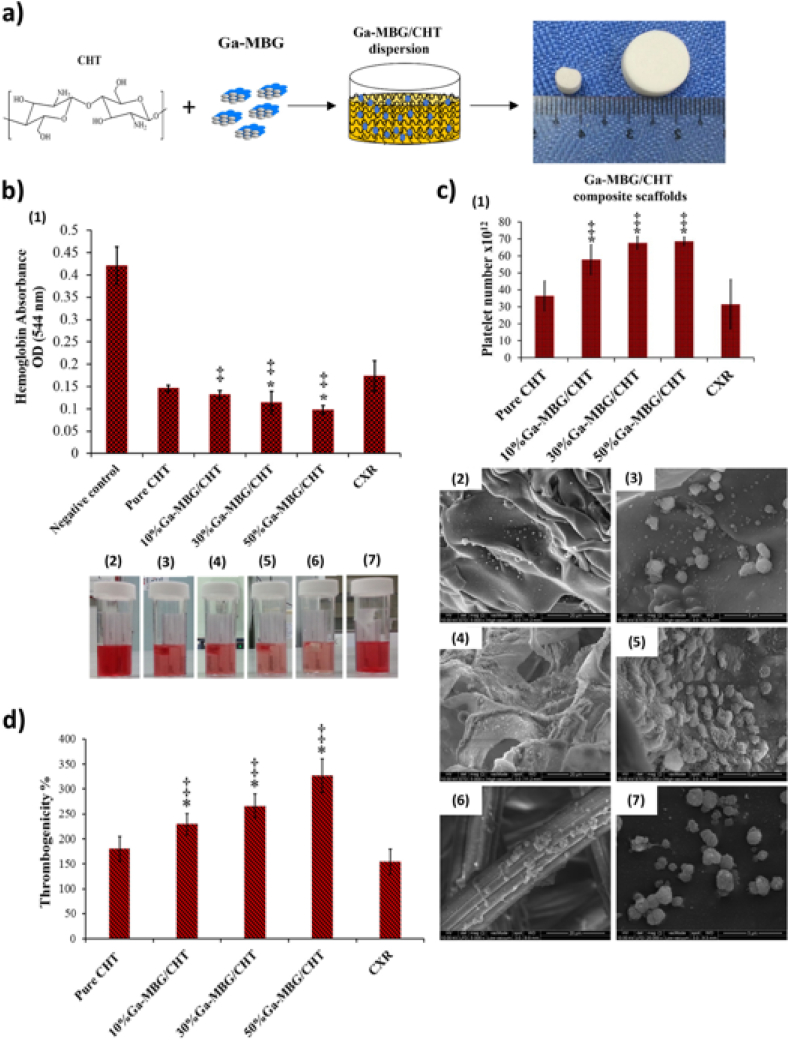
*a) Schematic illustration of fabrication process of chitosan and Ga containing MBGs composite by freeze drying, b) evaluation of blood clotting ability of fabricated composites compared to Celox*^*TM*^*Rapid gauze (CXR) and pure chitosan (pure CHT): (1) lower hemoglobin absorbance as an indicator of higher blood clotting rate, (2–7) images of hemoglobin leakage from negative control, pure chitosan, composites containing 10%, 30%, 50% bioactive glass and CXR respectively, c) evaluation of platelet adhesion after* 30 min *Incubation of platelet rich plasma(1), morphology of adhered platelets (2–7) in the same order, d) evaluation of thrombus formation after* 30 min Incubation with whole blood, (*,‡p < 0.05 compared with CHT and CXR, respectively). Reprinted with permission from ref. [17] *Copyright 2017 American Chemical Society.*

Pourshahrestani et al. [17] produced 1 mol % Ga doped MBGs with the EISA method (basic system: 80SiO_2_–15CaO–5P_2_O_5_) and combined them with chitosan scaffolds using a freeze-drying method (Fig. 7). The resulting composites increased the hemostatic performance of chitosan showing higher thrombus formation, blood clotting activity and improved amount of platelet adhesion in comparison to their Ga free counterparts. The composite material showed antibacterial activity against *E. coli* and *S. aureus* strains. The same authors developed a series of MBGs containing up to 3 mol% of gallium [41]. Gallium was found to increase blood coagulation and showed an antibacterial effect. The results also indicated that 1 mol % Ga_2_O_3_ addition could improve cytocompatibility and antibacterial properties without affecting the textural properties like surface area, pore size distribution, and pore volume. They also compared 1 mol % Ga doped MBGs against two commercial hemostats called Celox™ and QuickClot Advanced Clotting Sponge Plus™ [69], with the MBGs showing higher effectiveness than the commercial products. The Ga-doped MBGs can be thus considered suitable candidates for critical first aid treatments. Song et al. [121] synthesized 10 mol % Ga doped MBGs (SiO_2_–CaO–P_2_O_5_) and added them to orthodontic resins to prevent white spot lesions on enamel around orthodontic brackets. These white spot lesions are common to the treatment because bacteria such as *S. mutans* colonize the surrounding of the brackets after the treatment and produce acids like lactic acid which demineralize the enamel surface. The resin containing Ga doped MBG achieved in vitro remineralization of the enamel. The viability of *S. mutans* decreased as the gallium doped MBGs concentration in the resin increased.

MBGs can be utilized also as drug delivery carriers. Wang et al. [122] produced Ga-containing MBGs by loading Ga(NO_3_)_3_ into the mesoporous structure of MBGs instead of incorporating gallium in the glass structure. The Ga loaded MBGs were used for the production of 3D printed scaffolds with PCL matrix. Ion release behavior of the scaffolds was studied in PBS medium. However, the study did not assess the gallium release from the pores of the MBGs, thus making impossible to compare the data with results in the literature on gallium release from gallium containing MBGs. Moreover, the scaffolds showed a burst release of gallium during the first 3 days of incubation (200 μg/mL), followed by a linear gallium release up to 8 weeks (600 μg/mL). The authors demonstrated that Ga containing scaffolds have a potential to form a balance in bone homeostasis in infected bone defects using co-culturing with both bacteria (methicillin-resistant *S. aureus*) and osteogenic cells (MC3T3-E1). The scaffolds showed also promising results in the repair of infected bone defects in a rabbit model in vivo. Significantly lower amounts of bacteria and osteoclasts were found after treatment with Ga-loaded scaffolds [122].

The sol-gel method is also used to synthesize mesoporous bioactive glass nanoparticles (MBGNPs) for multifunctional biomedical applications. MBGNPs can be used in drug delivery applications and bioactive fillers in composite materials [111]. Kurtuldu et al. [123] synthesized up to 5 mol % Ga containing MBGNPs with a microemulsion assisted sol-gel method. Ga containing MBGNPs were based on the binary system 60SiO_2_–40CaO and exhibited bioactive behavior in-vitro. Additionally, ion release results indicated a relatively slow release of Ga ions in PBS medium. Ga containing MBGNPs exhibited antibacterial properties against *S. aureus* and *E. coli*, while showing no cytotoxicity towards MG-63 osteoblast cells.

## 4 Gallium in calcium phosphate bioceramics

Gallium can accumulate in bone, and in vivo studies have shown that bone fragments from gallium-treated animals are less soluble than untreated bones [65]. Later studies revealed that gallium ions are also clinically effective against bone resorption and for the treatment of osteoporosis and cancer-related hypercalcemia [43,125]. Gallium increases the calcium and phosphorus content of bone. Additionally, it blocks resorptive activity by inhibiting the vitamin D_3_-stimulated osteocalcin gene expression in osteoclast cells [62,126] without affecting the viability of osteoblast cells. Thus, incorporation of gallium into calcium phosphate bioceramics like HA or β-tricalcium phosphate (β-TCP) could create a synergetic effect in bone regeneration applications. The overview of gallium-containing bio-ceramics is summarized in Table 3. Melnikov et al. [127] first developed HA containing up to 11 mass % of gallium for bone regeneration applications. This study showed that gallium does not replace calcium in the HA crystals, and consequently produces no distortion in the framework of the hydroxyapatite matrix. Gallium exists in the HA crystals in the form of interstitial solid solution [127]. Ballardini et al. [128] showed that the incorporation of gallium in the HA structure acts as an effective antibacterial and antifungal agent when tested against yeast (*Candida albicans*), Gram-negative (*E. coli, P. aeruginosa*), and Gram-positive bacteria (*S. aureus*). The doping with gallium was effective in inducing an antibacterial effect against bacterial and fungus strains, without reducing the viability of human cells. Cassino et al. [129] studied gallium doped HA in *in-vivo* bone remodeling with Wistar rats, observing an improvement in the repair of the bone defects. The in-vivo study showed that gallium doped HA is an effective osteoinductive and osteoconductive agent, making it a promising candidate for bone regeneration applications.

**Table 3:** Gallium containing calcium phosphate (CaP) bioceramics.

Production Method	Composition/Phase (% mol)	Investigated Properties	Features	Ref.
Precipitation method	Hydroxyapatite (Ga_2_O_3_ up to 11.0 mass %)	Characterization of ceramic by SEM, EDX, TG, DTA, XRD, and thermomechanical properties.	Ga does not cause changes in the crystal structure of HA.	[127]
Precipitation method	Hydroxyapatite (molar ratio Ga/Ca) equal to 0.025, 0.05, and 0.1)	Characterization of ceramic by XRD, FTIR, BET, SEM, TG. Chemical composition and ion release behavior measured by ICP-OES. Cell viability assay using adipose-Derived Stem Cells (ASCs). Determination of the antibacterial activity of the scaffold against *S. Aureus, P. Aeruguinosa, E. Coli, and C. Albicans*.	Enhanced the antibacterial activity and osteoblast differentiation.	[128]
Precipitation method	Hydroxyapatite	In vivo biocompatibility study using albino Wistar male rats.	Improved the repair of bone defects.	[129]
Precipitation method and solid-state reaction	Hydroxyapatite (Ga content up to 0.35 mass%)	Characterization of ceramic by TEM, ICP-OES, XRD, FTIR, and NMR. Cell viability assay using BALB/c 3T3 cells. Antibacterial effect against *Pseudomonas fluorescens*.	Ga affected the crystal structure of HA. Showed antibacterial effect against *P. fluorescens*.	[136]
Solid-state reaction	Calcium Phosphate Cement, Ca_10.5-1.5x_Ga_x_(PO_4_)_7_	Characterization of ceramic by NMR, XRD, SEM. *In-vivo* sheep study.	Injectable. Increased new bone formation in osteoporosis sheep model.	[130]
Solid-state reaction	Calcium phosphate ceramics, (Ca + Ga)/P molar ratio of 1.515 and a Ga/Ca molar ratio in the 0–0.08 range.	Characterization of ceramic by XRD, solid-state NMR,	Improved mechanical properties. Showed a dose-dependent antiresorptive effect.	[131,137]
Solid-state reaction	β-TCP (up to 7.5 mol% Ga)	Characterization of ceramic by XRD and SEM. The cytocompatibility and in vitro osteoblastic differentiation were performed with mouse bone mesenchymal stem cells (mBMSCs). Osteoclast differentiation with RAW 264.7 cells.	Improved compressive strength. Suppressed *in-vitro* osteoclast differentiation.	[132]
Solid-state reaction	Calcium phosphate ceramics, (Ca + Ga)/P molar ratio of 1.515 and a Ga/Ca molar ratio in the 0–0.08 range.	Characterization of ceramic by XRD, solid-state NMR, SEM, and EDX. Cell viability assay using RAW 264.7 cell line. In vivo biocompatibility study using rabbits.	Ga release increased preferentially in the presence of osteoclasts. Showed a good interface between implant and newly formed bone in rabbit model.	[133]
Solid-state reaction	Calcium phosphate ceramics, (Ca + Ga)/P molar ratio of 1.515 and a Ga/Ca molar ratio in the 0–0.08 range.	Characterization of ceramic by XRD, solid-state NMR, SEM, and EDX. Cell viability assays using primary human osteoblasts and monocytes cells. In vivo bone reconstructive study using a murine bone defect-healing model.	Suppressed *in-vitro* osteoclast differentiation. Increased a new bone formation. Increased new bone formation in a rat model.	[61]
Solid-state reaction	β-TCP and Gallium containing phosphate glasses	Characterization of ceramic by XRD, SEM, and measurement of compressive stress of the scaffolds. In vitro osteogenic behaviors assessed by mBMSCs cell line. In vitro osteoclastic behavior evaluated using RAW 264.7 cell line.	Improved cell proliferation. Enhanced the late osteogenic markers. Suppressed osteoclast differentiation.	[134,135]

**Fig. 8:**
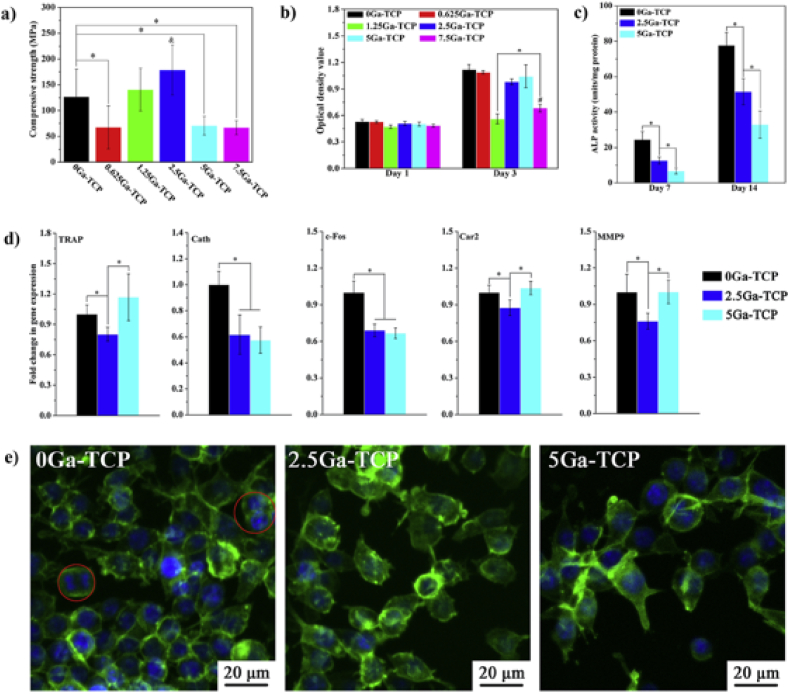
a) Compressive strength of Ga doped TCP bioceramics (*p < 0.05, &:significantly different from all the other samples), b) proliferation and c) ALP activity of mBMSCs after treatment of extracts of the samples, d) osteoclastic activity related genes expressions (*p < 0.05), e) fluorescence images of RAW 264.7 cells after 3 days of treatment with extracts of the samples (cytoskeleton and nuclei arestained in green and blue, respectively). Reproduced with permission from ref. [132]. *Copyright 2020 Elsevier.*

Gallium addition is also used in calcium phosphate ceramics. Janvier et al. [130] used gallium doping for β-tricalcium phosphate (β-TCP) [10]. Gallium in β-TCP ceramics of the composition Ca_10.5-1.5x_Ga_x_(PO_4_)_7_ substitutes one of the five calcium sites in the β-TCP lattice with random Ca/Ga distribution [130,131]. Qui et al. [132] showed that 2.5 mol % Ga addition to β-TCP was able to inhibit the phase transformation of β-TCP into α-TCP, which allowed for sintering of β-TCP at higher temperatures without phase transformation, leading to higher sintered densities and favorable mechanical strength of the produced β-TCP scaffolds. Gallium incorporation did not improve the proliferation of preosteoblast and ALP activity with respect to the Ga-free counterpart, while 2.5 mol % Ga addition to β-TCP (with release of 0.1 ppm Ga) exhibited lower expression of all the osteoclast-activity-related genes (TRAP, Cath, c-Fos, Car2, MMP9), as shown in Fig. 8. Gallium doped calcium phosphate bioceramics thus allow local delivery of gallium, which minimizes possible adverse effects of gallium for long-term oral treatments. Additionally, such local steady delivery increases the bioavailability of gallium [131]. This approach later led to the development of injectable apatite cements doped with up to 0.3 wt% gallium [133]. The injectable apatite cements are produced with two different precursors: gallium doped β-TCP and gallium doped calcium-deficient apatite (CDA). Different approaches to introducing gallium slightly improve the mechanical properties. Additionally, Ga-CDA showed local delivery of gallium ions. In-vivo studies showed an excellent interface between the implant surface and newly formed bone [61].

Strazic Geljic et al. [61] studied gallium-doped injectable apatite cements for in-vivo bone reconstructive applications, focusing on the gallium's inhibition effect on osteoclast cell differentiation. The authors studied gallium-doped CaP in bone defects generated in a rat model and showed that the local delivery of gallium through CaP had a positive impact on collagen synthesis and increased new bone tissue formation. The effect was linked to a decrease in osteoclast cell differentiation. Gallium thus can be considered an attractive additive to CaP biomaterials for reconstructive bone surgery.

He et al. [134,135] developed a hybrid system based on β-TCP and gallium doped phosphate bioactive glasses (GPGs) for producing bioceramic scaffolds via a solid-state reaction. The weight ratio of β-TCP to GPGs was 4:1 and the gallium amount in the phosphate glass system was up to 30 mol %. The addition of the gallium doped phosphate glasses to β-TCP promoted densification and increased the compressive strength of the scaffolds: 10 mol% gallium additions to phosphate glass resulted in a seven-fold increase in the compressive strength. The addition of 10–20 mol % gallium increased cell proliferation. Gallium doped scaffolds enhanced expressions of a later osteogenic marker and suppressed the expressions of osteoclast genesis related genes and the multinucleated RAW 264.7 cells. Therefore, these bioceramic scaffolds were considered a promising biomaterial for bone regeneration applications [134,135].

## 5 Gallium in coatings

The use of gallium in coatings on a variety of biomedical devices takes advantage of the antibacterial properties of gallium. Bacterial infections are one of the most critical reasons for the failure of implanted materials like prostheses. Oral and orthopedic implants could be colonized with bacteria, which could form a biofilm. Thereafter it could cause infection after implantation into the body, which is one of the most frequently faced post-surgery complications. Gallium ions show inhibition effect against *P. aeruginosa, Burkholderia cepacian complex, E. faecalis, methicillin resistant S. aureus, P. gengivalis, S. pyogenes, S. gordonii,* and *P. gingivalis* [138]. Mourino et al. [139] coated 45S5 Bioglass® scaffolds with sodium alginate crosslinked with gallium. Coated scaffolds exhibited antibacterial activity against *S. aureus* and improved mechanical properties (compressive strength) two-fold compared to uncoated scaffolds, without affecting the bioactivity provided by the 45S5 Bioglass®. Gallium-doped MBGs have been also used for coating titanium alloy (Ti6Al4V) substrates [140]. PCL and gallium doped MBG powder were mixed and applied on the surface of a Ti6Al4V alloy by dip coating. A homogenous, crack-free, and 1 μm thick coating was obtained, enabling apatite formation on the surface of the alloy substrate. Moreover, Stuart et al. [108,141] incorporated Ga into phosphate bioactive glass thin-films (Ga-PBG) to develop antibacterial activities and enhance osteogenesis potential for applications in next generation implant coatings. Ga-PBG coatings reduced the viability of *E. coli* and *S. aureus* bacteria compared to uncoated titanium.

Cochis et al. [142] modified the surface of titanium with gallium to prevent biofilm formation. The coating was applied by the anodic spark plasma (ASD) method. This electrochemical surface modification method uses a base electrolytic solution for the preparation of biomimetic coatings on dental implants [143]. To induce an antibacterial effect, gallium nitrate was added to the base solution. The antibacterial properties were evaluated on a dental implant in-vivo. Gallium-coated specimens showed the best inhibition ratio of metabolic activity (27–35%) of the human oral microbial flora, showing a stronger effect than silver coatings [142]. Cochis et al. [144] also studied gallium-coated titanium against multidrug-resistant *Acinetobacter baumannii*. *A. baumannii* is one of the most dangerous strains because it targets moist tissues exposed by surgical procedures [145]. Gallium coated samples showed good inhibition of biofilm formation with all three strains of *A. baumannii*. The antibacterial activity of the gallium coated sample was much stronger than that of the silver coated sample against *A. baumannii*. The study showed that slow and gradual release of gallium discourages the colonization and proliferation of *A. baumannii* on the implant surface. The coatings had no impact on the mechanical properties of titanium scaffolds.

Yamaguchi et al. [146] developed a surface coating of titanium with gallium via a simple hydrothermal ion-exchange process. First, the titanium surface was treated with NaOH, producing a nanostructured sodium hydrogen titanate layer (≈1 μm thick). Afterward, it was soaked in CaCl_2_ and GaCl_3_ solutions to ion-exchange Na with Ca and/or Ga, and the samples were heat treated at 600 °C for 1 h to prepare Ga-containing calcium titanate or gallium titanate phases on the surface. The coated titanium surface showed high antibacterial activity against *A. baumannii*. In addition to antibacterial properties, the bioactivity of the material was also improved. A hydrothermal ion-exchange process on porous sodium titanate allows sufficient penetration of Ga(NO_3_)_3_ solution into the surface structure [147]. Similar surface coating was applied to 3-D printed titanium implants [148]. Formation of a Ga-containing calcium titanate layer on the surface of titanium implants promoted apatite precipitation and enhanced the differentiation and mineralization of Saos-2 osteoblast-like cells [148]. Moreover, Chen et al. [149] developed a series of Mg–Ga layered double hydroxides (LDHs) nanosheet on alkaline treated (with NaOH) titanium surfaces. LDHs are composed of positively charged brucite-like layers [150]. Mg–Ga (molar ratio 1:1) LDHs coated titanium implants improved the local alkaline microenvironment (pH = 8.5), thus promoting osteogenic differentiation in an in-vivo study with Sprague Dawley rats.

Dong et al. [151] developed a different approach for coating the surface of titanium substrates. First, TiO_2_ nanotubes were prepared on the surface of titanium by electrochemical anodization. Afterward, the samples were soaked in a mixture of gallium nitrate and poly-dl-lactic acid (PDLLA) biodegradable polymer. This way, gallium nitrate was encapsulated with TiO_2_ nanotubes on the surface and provided local delivery of Ga^3+^. Ga coatings inhibited *S. aureus* and *E. coli* bacteria strains in in-vivo implantation and reduced the inflammatory response after surgery. Qiao et al. [152] synthesized SrTiO_3_ nanotubes on the surface of titanium by in situ growth hydrothermal method and soaked them in polydopamine (PDA) and gallium nitrate solution to form a PDA-Ga layer over the SrTiO_3_ nanotubes. The implants were tested against *E. coli* and *S. aureus*: gallium coated samples improved antibacterial activity, prevented the formation of bacterial colonies, and eliminated almost all bacteria within 24 h. Gallium coated titanium substrates maintained their antibacterial properties for up to 7 days culturing without reduction and retained approximately 72% of antibacterial activity for up to 14 days of culturing. The coatings possess high clinic translational potential, exhibiting multifunctional interfaces for orthopedic and dental implants.

Stan et al. [153] used the radio-frequency magnetron sputtering (RF-MS) method for coating titanium surfaces with Ga and Cu doped bioactive glass, using the FastOs®BG alkali-free glass composition. The glass coating did not show any cytotoxic behavior and served as an efficient antibacterial agent against *S. aureus* strain [153].

Ga^3+^ ions have been also homogeneously embedded in chitosan via an in-situ precipitation method utilizing the chelation ability of chitosan [154,155]. The complex was then coated on a stainless-steel substrate by electrophoretic deposition (EPD). Ga^3+^- chitosan complex showed a 90% higher bacterial inhibition effect against *E. coli* after 24 h of incubation, compared to pure chitosan. Besides the antibacterial properties, the coating showed non-toxic behavior in direct contact with MG-63 cells with about 70% cell viability even at the highest gallium concentrations (Ga^3+^:NH_2_ = 1:32, 1:16, 1:8, 1:4). The mechanical properties (hardness and critical load) of the polymer increased with decreasing gallium addition. However, a decrease of such properties was only observed at relatively high gallium additions due to restriction of mobility and deformation of the chitosan matrix [155].

## 6 Gallium in metallic alloys

Metals and alloys are widely used clinically as orthopedic implants [156,157]. Orthopedic implants occasionally face complications, such as failure after surgery due to bacterial infections. One of the strategies to prevent bacterial infection is an alloying biomaterial with antibacterial metals like Ag, Cu, and Zn [158]. Cochis et al. [159] added metallurgical gallium (up to 23 wt%) to titanium alloys to prevent biofilm formation on the surface of an implant, without enhancing the cytotoxicity of the alloy. The addition of 1–2 wt % gallium to titanium alloy had a significant antibacterial effect. At low gallium addition (1–2 wt %) no intermetallic phases were formed and gallium created a solid solution with α-titanium, providing a longer-lasting antibacterial effect than the coatings.

**Fig. 9:**
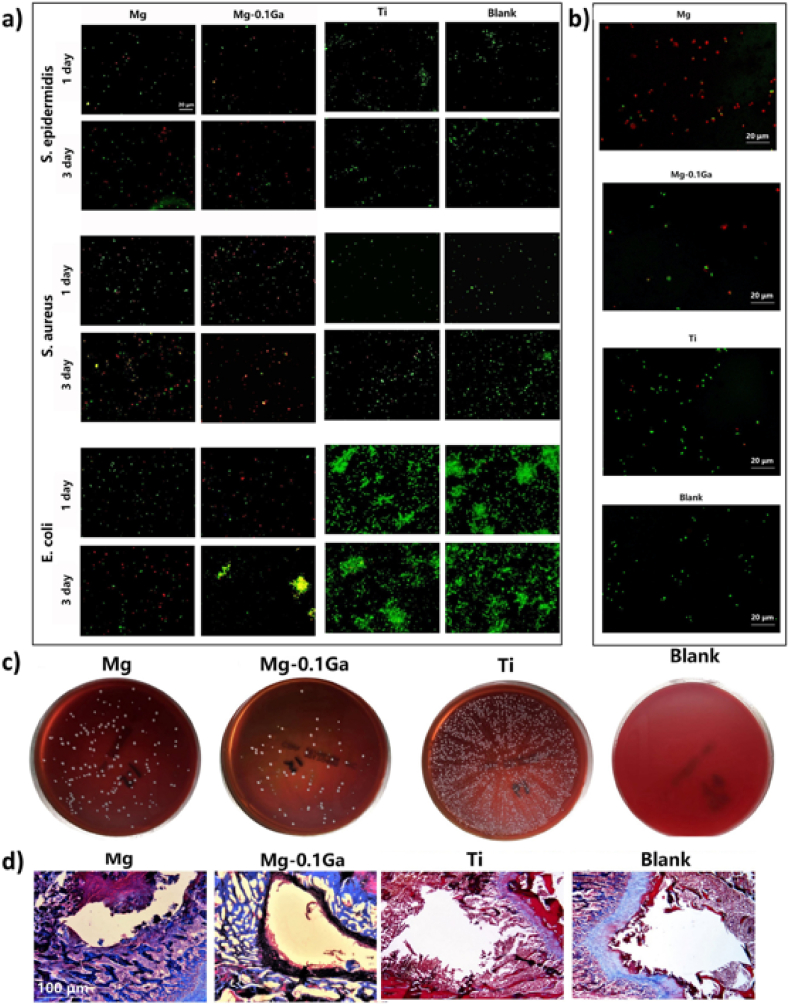
*Antibacterial activity of metal samples against S. epidermidis, S. aureus, and E.coli, a) fluorescence images of biofilm formation on Mg, Mg-0.1Ga, Ti and blank after 1 and 3 days (live and dead bacteria are stained in green and red respectively), b) live/dead staining of hMSCs in direct contact with metal materials after 24 h incubation, c)* in vivo *antibacterial activity of implanted metal rods retrieved from a mouse at day 5 in post-op time period, d) cross-sectional micrographs of implanted rods (mature bone tissue and fibrous tissue are stained in red and blue, respectively). Reproduced with permission from ref.* [166]. *Copyright 2019 Elsevier.*

Recently, a considerable body of literature has been emerging around magnesium alloys usage in biomedical applications due to their favorable mechanical properties and biodegradability [[160], [161], [162]]. He et al. [163] studied the effect of Ga addition (up to 7 wt% which is the maximum solid solubility) on the microstructure and mechanical properties of Mg–Ga alloys. Such Mg–Ga alloys have shown poor mechanical properties in as-cast form. Nevertheless, the mechanical properties of the Mg–Ga alloy could be improved by a plastic deformation process, such as extrusion. Mg_5_Ga_2_ phase was formed along with α-Mg phase, when the gallium content (3–7 wt%) increased in Mg–Ga alloys. Additionally, Ga addition has been shown to promote the dynamic recrystallization process of magnesium alloys during hot extrusion process [163,164]. Mg_5_Ga_2_ precipitated in the grain boundaries of α-Mg. Thus, Ga enhanced the mechanical properties of extruded Mg–Ga alloys by grain boundary strengthening and solid solution strengthening [163,165]. Moreover, Gao et al. [166] studied micro-allowing of Ga and/or Sr (0.1 wt%) in Mg. Due to the low concentration of alloying metals, there were no intermetallic phases formed (such as Mg_5_Ga_2_). The Mg alloy showed low in-vitro degradation. Moreover, the Ga-containing Mg alloy exhibited a lower cytotoxic behavior to human mesenchymal stem cells (hMSCs) compared to the alloy without Ga addition [166]. The addition of 0.1 wt % gallium to magnesium alloy showed antibacterial effect against *S. aureus*, *S. epidermidis* and *E. coli*. Moreover, the Mg alloy also inhibited *S. aureus* on the surface of implanted rods in an in-vivo rat model (Fig. 9) [166].

## 7 Conclusions

This review discussed the biological properties of gallium containing biomaterials. Gallium is used as a dopant in bioactive glasses, bio-ceramics, composites, coatings, and metallic alloys. Gallium as a therapeutic ion provides unique features to bioactive materials for a wide range of applications. It can bond with iron-binding proteins via shared physicochemical similarities with iron.

Gallium is the second metal after platinum being considered an anti-cancer drug. The anticancer and antibacterial activities of gallium are mainly associated with the competition of Fe^3+^ and Ga^3+^ for cellular uptake. Despite the anticancer activity of gallium, the ideal administration of Ga compounds still needs to be optimized. It is so far not largely explored, but gallium-containing bioactive materials have great potential for Ga delivery to treat cancer. Gallium-containing bioactive materials like glasses or bio-ceramics could be filled in the cavity created following bone surgery of tumorous growth and the controlled release of gallium could prevent further growth and proliferation of cancerous cells.

The osteogenic activity of gallium is associated with the destructive effect of gallium towards osteoclasts. Gallium was reported to reduce the resorption activity, differentiation, and formation of osteoclasts by non-cytotoxic mechanisms in a dose-dependent manner. Although the mechanism is unclear, the treatment with gallium shows an increased amount of calcium and phosphate content of bone, which leads to higher resistance to bone resorption. Gallium also shows potential for wound healing. It can be used not only at a later stage (e.g., due to its antibacterial properties to treat inflammation), but also in the very early phase of wound healing due to its hemostatic function via activating intrinsic coagulation pathways. Moreover, gallium containing biomaterials show promising results in suppression of drug-resistant bacteria, with various degrees of effectiveness, depending on the composition of the biomaterial and type of bacteria. Ga can be therefore used to improve a broad range of biomaterials, from coatings, through glasses and ceramics, to metal alloys for a variety of applications.

Gallium incorporation in various types of bioactive glasses and bio-ceramics is attractive for coating applications. Due to the lack of coherency concerning the chemical composition, direct comparison of biological assessments is difficult. However, as shown throughout this review, gallium containing biomaterials exhibit genuine biological effects in the contexts of in vitro and in vivo studies. Taking into consideration the experimental results summarized in this review, further systematic investigations are required concerning the structural role of gallium in different bioactive materials, in order to control its sustained delivery to establish long-term therapeutic efficacy and biological activity of gallium containing bioactive materials.

## Declaration of competing interest

The authors declare that they have no known competing financial interests or personal relationships that could have appeared to influence the work reported in this paper.
